# Differentiated LUHMES Cells as a Model to Investigate Neurotropic Arboviruses and Evaluate Host-Directed Therapeutics

**DOI:** 10.3390/microorganisms14091944

**Published:** 2026-09-02

**Authors:** Lorreta Aboagyewa Opoku, Stephanie V. Trefry, Maame Konadu, Jonathan Ontivero Sanchez, Alison Gomeiz, Shannon D. Walls, Michael D. Barrera, Dylan Valerio Scarton, Rémi Veneziano, Mariaelena Pierobon, Elsa Ronzier, Aarthi Narayanan

**Affiliations:** 1 School of Systems Biology, College of Science, George Mason University, Manassas, VA 20110, USA; 2 Biology Department, College of Science, George Mason University, Fairfax, VA 22030, USA; 3 Center for Applied Proteomics and Molecular Medicine, George Mason University, Manassas, VA 20110, USA; 4 Biomedical Research Laboratory, George Mason University, Manassas, VA 20110, USA; 5 Institute for Advanced Biomedical Research, George Mason University, Manassas, VA 20110, USA; 6 Interdisciplinary Program in Neuroscience, College of Science, George Mason University, Fairfax, VA 22030, USA; 7 Department of Bioengineering, George Mason University, Fairfax, VA 22030, USA

**Keywords:** LUHMES, neuronal model, Alphavirus, Flavivirus, Omaveloxolone

## Abstract

Arthropod-borne viruses such as Alphaviruses and Flaviviruses are the causative agents of severe human disease, highlighted by fatal encephalitis and neurological sequelae in survivors. The lack of FDA-approved vaccines and therapeutics that can prevent or treat these infections results in a significant global disease burden. An important unmet need to address this capability gap is the need for affordable, scalable, clinically relevant human-based neuronal models to study neuroinvasive viruses and evaluate therapeutic options. Here, we described the application of a human neuronal precursor cell model, LUHMES (Lund human mesencephalic) cells, that can be differentiated into dopaminergic midbrain neurons and used to study virus infections. In this study, we demonstrated the susceptibility of LUHMES cells to infection by three arthropod-borne neurotropic viruses: Venezuelan equine encephalitis virus, dengue virus serotype 2, and West Nile virus. We also demonstrated how the model may be applied to evaluate potential therapeutic options using an FDA-approved small molecule, Omaveloxolone. Finally, we analyzed host cell responses to infection and treatment using gene expression and phospho-signaling analyses. These findings highlight the value of this model to interpret the pathogenic mechanisms of neurotropic viral infections and evaluate potential therapeutic intervention strategies in a clinically relevant in vitro human neuronal model.

## 1. Introduction

The geographical distribution of arthropod-borne RNA viruses is widespread, resulting in millions of people affected yearly [1,2,3,4]. Left untreated, infections caused by viruses such as Alphaviruses and Flaviviruses initiate human disease that can progress into the central nervous system (CNS), resulting in death or long-term sequelae in survivors [5,6,7]. Several of these viruses, such as Venezuelan equine encephalitis virus (VEEV), West Nile Virus (WNV), and dengue virus (DENV), cause encephalitic pathologies resulting in debilitating symptoms throughout affected individuals’ lives [8,9,10]. Virus-induced damage to neurons is in part causally linked to several clinical manifestations, including Parkinson’s-like disease, encephalitis, acute flaccid paralysis, cognitive, sensory, and motor disorders [3,6,11]. There is a well-recognized lack of FDA-approved therapeutics or vaccines to treat or prevent infection for several of the above-mentioned arthropod-borne viruses. A critical capability gap that contributes to the absence of a robust therapeutic discovery pipeline is the lack of clinically relevant, human-based in vitro models to investigate neurological mechanisms involved in the host response to viral infection and resulting pathology [2,12,13,14]. This capability gap can be addressed by deploying human-based neurological models that recapitulate virus–host interactions in the nervous system. Human-induced pluripotent stem cell (hiPSC)-derived neurons and mouse-derived neuronal cells physiologically resemble human cells. While considering such primary cells, it is also essential to remember that these cells reach senescence after a few passages, if proliferative, which results in limited cell numbers and loss of phenotype over time. Additionally, donor-to-donor variability is common in the context of primary cells, which further compounds variability in experimental outcomes [15,16]. Lastly, hiPSC-derived cells are developed from stem cells and hence retain fetal characteristics that can impact downstream applications.

Lund human mesencephalic (LUHMES) cells may offer an alternate neuronal cell option to hiPSC, primary cells, and conventional immortalized cell models. LUHMES cells are a subclone of tetracycline-controlled MESC2.10 cells, which are human embryonic neuronal precursor cells [17,18]. Due to the expression of a tetracycline-regulatable (Tet-off) *v-myc* transgene, LUHMES cells can proliferate rapidly and be differentiated into post-mitotic neurons within a short timeframe with the addition of tetracycline, glial cell-derived neurotrophic factor (GDNF), and dibutyryl cAMP [19,20]. These cells offer features that align with human neurons, without the genetic alterations of immortalized cell lines, to provide a neuronal-like phenotype [21,22,23,24]. LUHMES are accessible to a wide range of researchers, as their cost is comparable to immortalized cell lines; they stably proliferate up to 25 passages and have a short population doubling time of 30–40 h [25]. Their scalability, reproducibility, rapid differentiation, cost-effectiveness, and neuronal-like phenotype make them ideal candidates to study neurotropic viruses. Regarding application in human viral infections, studies have demonstrated the utility of LUHMES cells with Herpes simplex virus 1 (HSV-1), which is seen to support virus replication [14,26,27,28].

In this work, we demonstrate the potential application of this cell type to better understand arthropod-transmitted virus infection of neurons. The results presented in this study demonstrate the susceptibility of this cell type to neurotropic viruses using attenuated VEEV TC-83, DENV-2, and WNV as prototype pathogens. Our studies also show the responsiveness of this cell type to a host-directed therapeutic strategy. Lastly, our studies show host responses to the infection and treatment strategy in a limited gene expression context and phospho-signaling responses. Collectively, the data presented here provide a foundation for expanded utilization of this model to understand the pathological outcomes of virus infections of neurons in a clinically relevant context, and to evaluate host-based therapeutic intervention strategies.

## 2. Materials and Methods

### 2.1. Cells

Lund human mesencephalic (LUHMES) cells (ATCC, Cat # CRL-2927, Manassas, VA, USA) were purchased from the American Type Culture Collection (ATCC, Manassas, VA, USA) and cultured in their neural progenitor state (undifferentiated) in Dulbecco’s Modified Eagle Medium (DMEM) and Ham’s F-12 Nutrient Mixture (1:1) (DMEM: F-12; ATCC, Cat # 30-2006) supplemented with 1% N2 (Gibco, Thermo Fisher Scientific, Cat # 17502-048, Waltham, MA, USA). Prior to studies, flasks and/or plates were pre-coated with 50 µg/mL of poly-L-ornithine (Sigma-Aldrich, Cat # P-3655, St. Louis, MO, USA) overnight at room temperature or for 2 h at 37 °C. Following incubation with poly-L-ornithine, plates/flasks were coated with 1 µg/mL of fibronectin (Sigma-Aldrich, Cat # F-1141) for either 2 h at 37 °C or overnight at room temperature. Prior to seeding cells, plates/flasks were rinsed with sterile water three times and allowed to dry. From neuronal progenitor state to differentiation into mature neurons, DMEM F-12 media was supplemented with 1 mM of cAMP (Santa Cruz, Cat # 16980-89-5, Dallas, TX, USA), 1 μg/mL of Tetracycline (Sigma-Aldrich Cat # T-7660), 2 ng/mL of GDNF (Gemini-bio, Cat # 300-121P, West Sacramento, CA, USA), and 1% of N-2 supplement. Cells were either undifferentiated (undiff.), differentiated for 3 days (3d diff.), 6 days (6d diff.), or 14 days (14d diff.) before being used for experiments.

Vero cells (ATCC, Cat # CCL-81) were cultured in DMEM supplemented with 10% heat-inactivated fetal bovine serum (HI-FBS; Thermo Fisher Scientific, Cat # A52568-01, Waltham, MA, USA), 1% L-glutamine (Gibco, Cat # 25030-081), 1% NEAA (Gibco, Cat # 11140-050), and 1% penicillin/streptomycin (Corning, Cat # 30-002-Cl, Corning, NY, USA). All cells were cultured in humidified incubators at 37 °C and 5% CO_2_.

### 2.2. Viruses

Venezuelan equine encephalitis virus vaccine strain TC-83 (VEEV TC-83) was derived from an infectious clone kindly provided by Ilya Frolov (University of Alabama at Birmingham) [29]. A plasmid containing the infectious cDNA of VEEV TC-83 was used for in vitro transcription and transfection into Vero cells. The rescued virus was passaged once in Vero cells and identity verified via sequencing. Virus stock was titrated by plaque assay on Vero cells. West Nile virus (WNV; MX H 442) and dengue virus serotype 2 (DENV-2; New Guinea C) were obtained from BEI resources. WNV and DENV-2 were amplified according to BEI’s product information sheets.

### 2.3. Virus Titration

Plaque assays were performed on Vero cell monolayers, plated to reach ~90% confluency in 12-well plates (Greiner Bio-One Cellstar, Cat # 665180, Monroe, NC, USA), infected for an hour, and overlaid, fixed, and counted as described in [30] after 48 h of incubation for VEEV TC-83 virus and 72 h of incubation for WNV. For DENV-2, live cells were stained with 0.33% neutral red (Sigma-Aldrich, Cat # N2889, St. Louis, MO, USA) mixed with 1% melted agarose to a 0.05% final concentration 96 h after incubation, then re-incubated for an additional 20 h, after which plaques were counted.

### 2.4. Western Blot

LUHMES cells were seeded to reach ~80% confluence in 12-well plates (Greiner Bio-One Cellstar, Cat # 665180). At the time points and differentiation states indicated in the figures, cells were washed with PBS (Thermo Fisher, Cat # 10010023) and lysed with Blue Lysis Buffer as described in [2]. All lysates were normalized to protein concentration, and 5–15 µg protein was loaded into a Bis-Tris Mini Protein Gel, 4–12%, 1.0–1.5 mm (Thermo Fisher, Cat # NP0321BOX) at 100 V for 60–90 min in 1X NuPAGE^TM^ MOPS SDS Running Buffer (Thermo Fisher, Cat # NP0001). After protein separation, proteins were transferred to a PVDP membrane at 4 °C for one hour in 1X NuPAGE^TM^ Transfer Buffer (Thermo Fisher Scientific, Cat # NP0006). The membrane was blocked for one h at RT in 1X BSA (Thermo Fisher, Cat # ZJ402850). Following the blocking step, membranes were incubated at RT for two hours or overnight at 4 °C with primary antibodies prepared in 1X BSA at the dilutions indicated below. Primary antibodies were washed three times with TBST and then incubated with HRP-conjugated secondary antibodies at the indicated dilutions for one hour at RT. Finally, membranes were washed three times with TBST and once with TBS prior to imaging using SuperSignal^TM^ West Femto Maximum Sensitivity Substrate (Thermo Fisher, Cat # 34094) and the Bio-Rad ChemiDoc Imager (Hercules, CA, USA). Band intensity was analyzed using ImageJ software (NIH, Bethesda, MD, USA; v.1.53q).

### 2.5. Replication Kinetics

Vero and LUHMES (undiff, 3d diff, 6d diff, and 14d diff) cells were seeded to achieve ~50–60% confluent monolayers overnight in 12-well plates. Cell monolayers were mock-treated (PBS) or infected with VEEV TC-83 at an MOI of 0.01, 0.1, or WNV, or DENV-2 at an MOI of 1 or 5. Following a one-hour incubation, the virus inoculum was removed, washed once, complete media were added, and plates were incubated in a humidified incubator at 37 °C and 5% CO_2_. For VEEV TC-83, supernatants from triplicate wells were collected at 0, 6,12, 24, 48, and 72 hpi, and the volume was replaced with complete media. Supernatant samples were stored at –80 °C until further analysis. Infectious titers were quantified by plaque assay on Vero cells. The same methodology was used to evaluate WNV and DENV-2 replication kinetics at 0, 24, 48, and 72 hpi.

### 2.6. Cytotoxicity Assay

Omaveloxolone (OMA) was purchased from MedChemExpress (Cat # HY-12212, Monmouth Junction, NJ, USA) and resuspended in DMSO (Thermo Fisher Scientific, Cat # 194474) to generate 10 mM stocks. To assess cytotoxicity, differentiated LUHMES cells were seeded to reach ~80% confluence in 12-well plates. Cells were untreated, DMSO-treated, or OMA-treated for 24 h, after which a 50/50 dilution of media and CellTiter-Glo reagent was used to treat cells for 10 min, and 100 μL supernatant was transferred to white 96-well plates (Thermo Fisher Cat # 165306), and cell viability was assessed utilizing the CellTiter-Glo^®^ Luminescent Cell Viability Assay (Promega, Cat # G7573, Madison, WI, USA) per manufacturer’s instructions. Cytotoxicity was assessed in a 5-point dilution curve at concentrations indicated on graphs to determine 50% cytotoxic concentration (CC_50_) using GraphPad Prism.

### 2.7. Infection and Treatment Assessment

For the evaluation of the efficiency of OMA, cells were either pretreated with OMA or dimethyl sulfoxide (DMSO) at 0.001 μM for an hour, inoculum removed, and or infected with VEEV TC-83 or WNV or DENV at MOIs indicated in the figure legend for an hour, the inoculum removed, and post-treated at the same concentrations or replaced with fresh complete media for untreated, or uninfected mock controls, and supernatants collected at the indicated time points. Samples were titrated on Vero cells as described previously. For the time-of-addition assay, 3d diff and 6d diff LUHMES cells were infected (MOI = 0.1) with VEEV TC-83 luciferase-tagged virus or mock-treated, incubated for an hour, and treated with OMA after infection at 0, 2, 4, 6, and 8 h post-infection. After 24 h of incubation, Nano-Glo Luciferase Assay reagent (Promega, Cat # N1130) was added following the manufacturer’s protocol and read on the Promega GloMax reader.

### 2.8. Immunofluorescence

LUHMES cells were seeded to reach ~50% confluence in 24-well glass-bottom plates (Cellvis, Cat # P24-0-N, Mountain View, CA, USA). Cells were mock-treated (PBS) and infected with VEEV TC-83 as described in Section 3. Following the incubation period, cells were washed once with PBS and fixed with 4% paraformaldehyde (Thermo Fisher, Cat # A5818101) for 10 min at room temperature (RT). Cells were then washed three times with PBS, permeabilized with 0.25% Triton X-100 (Thermo Fisher Scientific, Cat # HFH10) for 10 min at RT. Following permeabilization, cells were washed three times with PBS and blocked with 3% BSA (Thermo Fisher Scientific, Cat # 37525X3, Waltham, MA, USA) for 45 min at RT. Cells were then incubated with primary antibodies (Table 1) in 3% BSA for 2 h at RT, washed three times with PBS, and incubated with secondary antibodies (Table 1) in 3% BSA at RT for 1.5 h at dilutions indicated in the table below. Images were acquired using either an Image Xpress Micro Confocal Imaging System (Molecular Devices, San Jose, CA, USA) or a Nikon Instrument Inc. (Melville, NY, USA) widefield fluorescence microscope. Objectives used for representative images in the figures are indicated in the figure legends.

**Table 1:** Antibodies for Western blot (WB) and immunofluorescence (IF).

Antibodies	Vendor	Catalog #	Dilution Used for IF or WB
Anti-beta-III tubulin	R&D systems (Minneapolis, MN, USA)	MAB1195	1:200 (IF) 1:1000 (WB)
Anti-GAPDH monoclonal antibody loading control (GA1R), HRP	Thermo Fisher	MA5-15738-HRP	1:5000 (WB)
Anti-Venezuelan equine encephalitis virus NSP1 (HL1472)	Thermo Fisher	MA547057	1:1000 (WB)
Anti-Venezuelan equine encephalitis virus NSP2 (8A4B3)	Kerafast (Vector Laboratories, Boston, MA, USA)	EULO15	1:1000 (WB)
Anti-Venezuelan equine encephalitis virus NSP3 (HL1502)	Thermo Fisher	MA547070	1:1000 (WB)
Anti-Venezuelan equine encephalitis virus E2 (VEEV-57)	bioXcell (Lebanon, NH, USA)	BE0435	1:200 (IF)
Alexa Fluor^TM^ 488 phalloidin	Thermo Fisher	A12379	1:600 (IF)
DAPI (Hoechst 33342)	Thermo Fisher	I34406	1:600 (IF)
Alexa Fluor^TM^ 568 donkey anti-mouse IgG	Thermo Fisher	A10037	1:2500 (IF)
Alexa Fluor^TM^ 488 donkey anti-rabbit IgG	Thermo Fisher	A21206	1:2500 (IF)
Goat anti-mouse IgG secondary antibody, HRP	Thermo Fisher	32430	1:5000 (WB)
Goat anti-rabbit secondary antibody, HRP	Thermo Fisher	32460	1:5000 (WB)

### 2.9. RNA Extraction

LUHMES cells were seeded to reach ~80% confluency in 6-well plates. Cells were differentiated for 6 days and either mock-treated (PBS) or OMA-treated (0.001 µM) and/or VEEV TC-83-infected at an MOI of 0.1 for 16 h. Cells were washed once with PBS, and RNA was extracted using the RNeasy Protect Mini Kit per the manufacturer’s instructions. Intracellular RNA was extracted using the RNeasy Mini Kit (QIAGEN, Cat # 74106, Hilden, Germany) per the manufacturer’s instructions. RNA yield and purity were analyzed via NanoDrop to ensure high-quality RNA for downstream analysis and stored at −80 °C until further analysis.

### 2.10. Neurotoxicity Gene Expression Arrays

Extracted RNA, as described previously, was converted to cDNA and added to Human Neurotoxicity RT^2^ Profiler PCR Array plates (QIAGEN, Cat # 330231, Germantown, MD, USA). The plates were analyzed on the RNA UltraSense^TM^ One-step Quantitative RT-PCR System (Thermo Fisher Scientific, Waltham, MA, USA). Quantification of the expression of neurotoxicity-associated genes was calculated using the QIAGEN GeneGlobe Data Analysis Center.

### 2.11. qRT-PCR Assay

Extracted RNA, as described previously, was quantified using the RNA UltraSenseTM One-step Quantitative RT-PCR System (Applied Biosystems, Thermo Fisher Scientific, Waltham, MA, USA). Viral RNA detection was performed with the Verso 1-Step qRT-PCR Rox kit (Thermo Fisher, Cat # AB4101C) using primers and probes defined below (Table 2). A standard curve of known VEEV TC-83 RNA was used, and absolute quantities based on threshold cycle relative to the standard curve were calculated in the StepOne software (version v2.3). PCR equation, y = mx + b, −3.559x + 40.719 = y, R2 = 0.999, Eff% = 90.969, Threshold = 0.026154. For independent gene expression analysis, EREG and CASP7 (Thermo Fisher Cat #, Hs00914313_m1 and Hs00169152_m1) samples were normalized to GAPDH (Thermo Fisher Cat #, Hs02786624_g1) and quantified using the 2^−ΔΔCt^ method relative to mock controls [31].

**Table 2:** Primers and Probes.

Name	Nucleotide Sequence
**nsP1—forward primer**	CTGACCTGGAAACTGAGACTATG
**nsP1—reverse primer**	GGCGACTCTAACTCCCTTATTG
**nsP1—probe**	TACGAAGGGCAAGTCGCTGTTTACC

### 2.12. Reverse Phase Protein Array (RPPA)

LUHMES cells were seeded to reach ~80% confluency overnight in 6-well plates. Cells were either mock-treated (PBS) or infected with VEEV TC-83 (MOI 0.1) and treated with DMSO or OMA (0.001 µM) for the RPPA study. Cells were washed twice with PBS and lysed using Tissue Protein Extraction Reagent (TPER; Thermo Fisher, Cat # 78510) and 2X SDS-PAGE Sample Buffer (Thermo Fisher, Cat # LC2676) mixed with 2.5% beta-mercaptoethanol (Gibco, Thermo Fisher Scientific, Cat # 21985023, Waltham, MA, USA) for a final concentration of 500 μg/mL and stored at −80 °C until further handling. Cell lysates were immobilized onto nitrocellulose-coated glass slides (Grace Bio-Labs ONCYTE, Cat # GBL505170, Bend, OR, USA) using a Quanterix 2470 Arrayer (Quanterix, Billerica, MA, USA) as technical replicates (n = 4). Protein was quantified with Sypro Ruby Protein Blot Stain (Thermo Fisher, Cat # S11791) per the manufacturer’s instructions. The remaining arrays were first treated with Reblot Antibody Stripping solution (Chemicon, MilliporeSigma, Cat # 2502, Burlington, MA, USA) for 15 min at RT, followed by two washes with PBS, and incubated for at least 4 h in I-Block. Using an automated staining system, arrays were probed with 3% hydrogen peroxide (Agilent Technologies, Cat # K1500, Santa Clara, CA, USA), a biotin blocking system, and an additional serum-free protein block to reduce background signal. Each array was probed with optimized primary antibody dilutions (Supplementary Table S1). Biotinylated anti-rabbit and anti-mouse secondary antibodies (Vector Laboratories, Cat # BA-1000 and BA-2000, 1:7500, Newark, CA, USA) coupled with a commercially available tyramide-based avidin/biotin (Vector Laboratories, Cat # BA-2000) amplification system were then used to quantify signaling molecules within each sample. Antibody- and Sypro Ruby-stained slides were imaged on a Tecan PowerScanner (Tecan, Mannedorf, Switzerland) laser scanner, and images were analyzed using the commercially available MicroVigene Software (Version 5.1.0.0). Data were analyzed in JMP software and normalized before clustering. Unsupervised hierarchical clustering was performed in JMP Pro v17 using Ward’s normalization method for the heat map.

### 2.13. Statistical Analysis

Statistical analyses, as indicated in figure legends, were calculated from an average of three independent experiments using GraphPad Prism (version 10.5.0). Statistical significance was defined as * *p* < 0.05, **p < 0.01, *** *p* < 0.001, **** *p* < 0.0001, and *p* = ns (not significant), Samples with statistics were analyzed based on the average of n > or = 3 biological replicates.

## 3. Results

### 3.1. Differentiation of LUHMES Cells and Verification of Cell Type-Specific Markers

**Figure 1:**
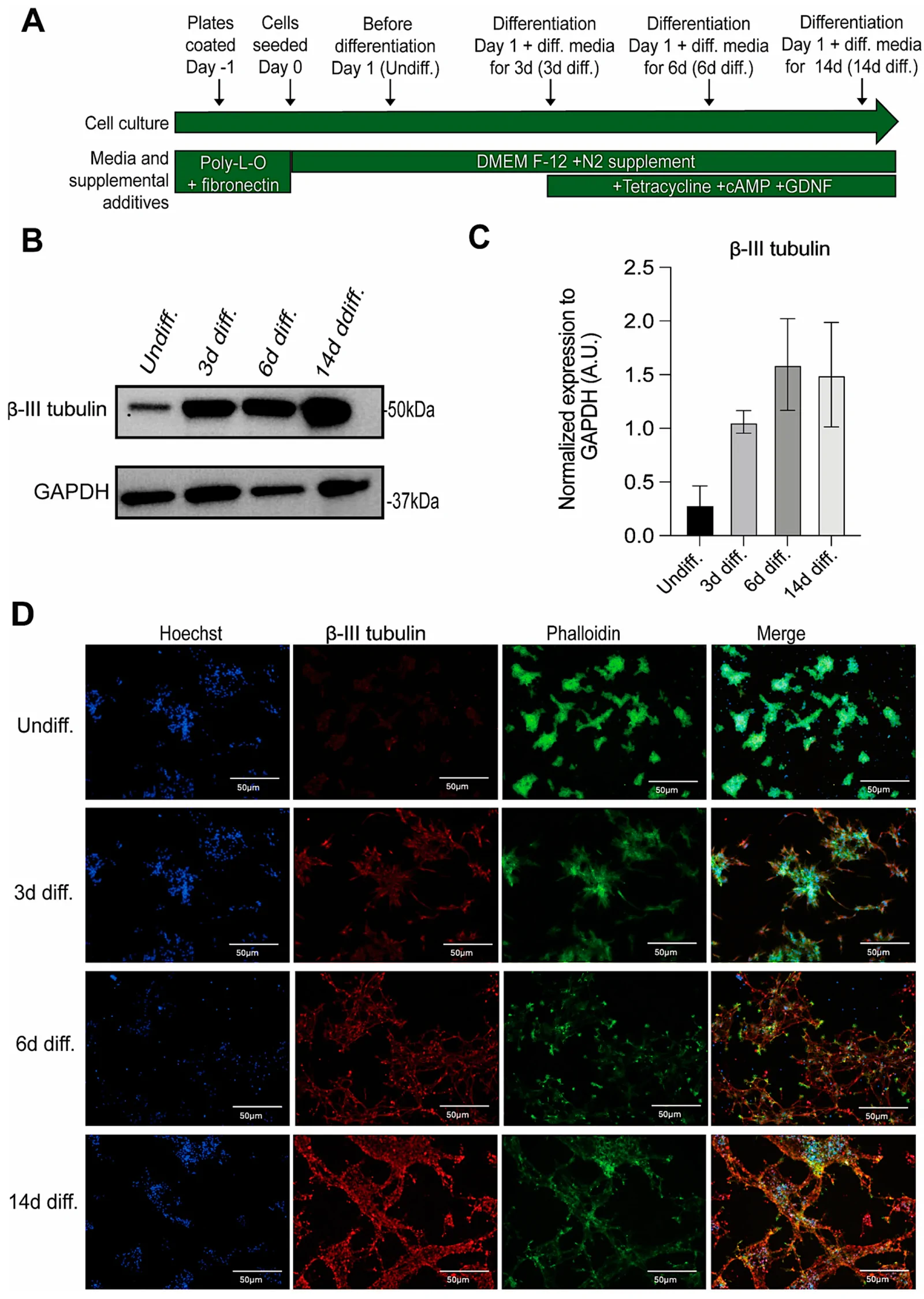
Differentiation of LUHMES cells and validation of neuronal maturation. (**A**) Graphical overview of the differentiation of LUHMES cells. (**B**) Representative Western blot against beta-three tubulin (β-III) and GAPDH in undifferentiated (Undiff.), 3-day (3d diff.), 6-day (6d diff.), and 14-day (14d diff.) differentiated LUHMES cells. (**C**) Average normalized β-III tubulin protein expression using GAPDH as a loading control, using ImageJ for band intensity analysis (n = 2). (**D**) Representative immunofluorescence images of Undiff., 3d diff., 6d diff. and 14d diff. LUHMES cells were stained with β-III tubulin; Hoechst staining was included as a nuclear marker, and phalloidin (filamentous actin marker) at 10x magnification.

Differentiation of LUHMES cells was carried out following previously published methodologies [20]. The differentiation strategy is illustrated in the schematic (Figure 1A). Briefly, undifferentiated LUHMES cells were cultured in differentiation media containing tetracycline, cyclic adenosine 3′,5′-monophosphate (cAMP), and glial cell line-derived neurotrophic factor (GDNF) for three (3d diff.), six (6d diff.), and 14 days (14d diff.) [20,26]. A neuron-specific marker, beta-three tubulin (β-III tubulin), was selected to evaluate neuron-specific protein expression between undifferentiated and differentiated cells by Western blot (Figure 1B and Figure S1A). A time-dependent increase in β-III tubulin was observed in 3d, 6d, and 14d differentiated LUHMES cells compared to undifferentiated cells, verifying their progressive differentiation. Quantification of band intensity showed an increase in β-III tubulin in all differentiated LUHMES cells when compared to undifferentiated LUHMES cells (Figure 1C).

Analysis by immunofluorescence with β-III tubulin antibody of differentiated LUHMES cells showed characteristic neuronal morphology with clearly defined structural components such as cell body, axonal terminals, and axon shaft with branching neurites (Figure 1D and Figure S1B). Paralleling our results from the Western blot analysis, an increase in β-III tubulin was observed as cells increased in differentiation age. Hoechst staining was included as a nuclear marker, and phalloidin (filamentous actin marker). Altogether, this strategy produced differentiated, mature neuronal-like cells that reliably expressed the neuron-specific marker, β-III tubulin [17].

### 3.2. Susceptibility and Replication of Neurotropic Arboviruses in LUHMES Cells

**Figure 2:**
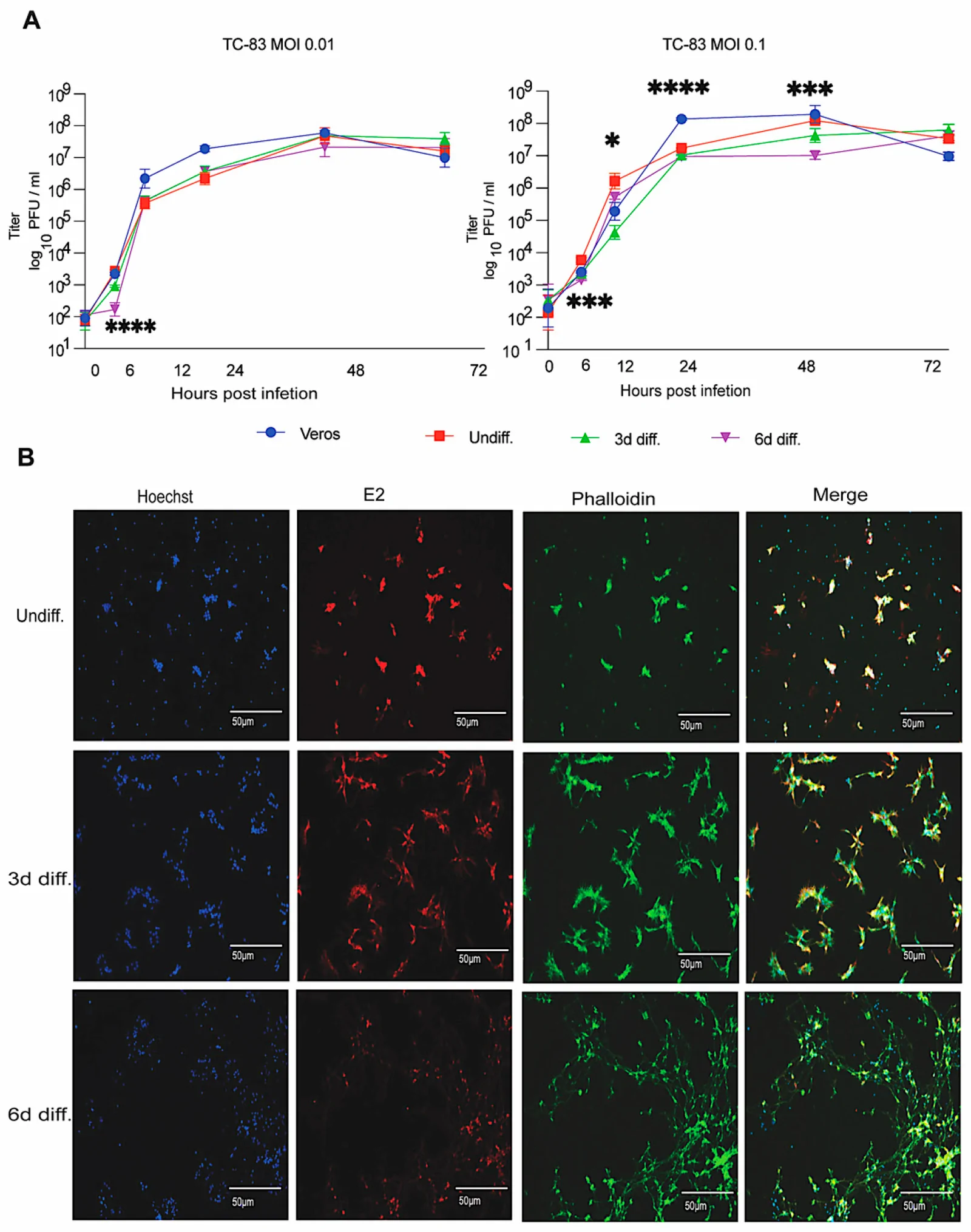
Susceptibility and replication kinetics of Venezuelan equine encephalitis virus, vaccine strain TC-83 (VEEV TC-83) in Vero cells and differentiated/undifferentiated LUHMES cells. (**A**) Replication kinetics of Vero cells, undifferentiated (undiff), 3-day (3d diff), and 6-day (6d diff) differentiated LUHMES cells infected with VEEV TC-83 at an MOI of 0.01 and 0.1 at 0, 6, 12, 24, 48, and 72 h post-infection (hpi). Values are the average of three biological replicates ± standard deviation. Averages of each time points were analyzed via one-way ANOVA relative to undifferentiated cells. * *p* < 0.05, *** *p* < 0.001, **** *p* < 0.0001. (**B**) Representative immunofluorescence images at 10x magnification of undifferentiated and 3d and 6d differentiated LUHMES cells infected with VEEV TC-83 at an MOI of 0.1 at 24 hpi and stained against VEEV E2 at 24 hpi with Hoechst staining as a nuclear marker, and phalloidin (filamentous actin marker). Scale bars represent 50 µm.

To investigate the susceptibility and replication of arthropod-borne viruses, differentiated and undifferentiated LUHMES cells were first infected with the VEEV TC-83 strain. Vero cells were maintained alongside as a positive control cell type for infection. Cells were infected with VEEV TC-83 at a multiplicity of infection (MOI) of 0.01 and 0.1, and sample supernatants were collected at 0, 6, 12, 24, 48, and 72 h post-infection (hpi). Viral infection kinetics were subsequently quantified using the supernatants via plaque assay at all time points mentioned (Figure 2A).

Regardless of MOI and cell type, a significant increase in infectious titer was observed within 12 hpi. Peak viral titers were reached in all cells between 24 and 48 hpi at the MOI of 0.1 and 0.01. The scale of replication was dependent on the differentiation period, where undifferentiated cells had the highest viral titers, followed by 3d and then 6d differentiated cells [32]. These results support previous studies where maturation of neuronal cells impacts permissiveness, yielding lower infectious titers in mature neurons relative to immature neurons [32,33,34].

We expanded these initial results with two other relevant neurotropic Flaviviruses, West Nile virus (WNV) and dengue virus serotype 2 (DENV-2) (Figure S2). Similar to the approach with VEEV TC-83, undifferentiated and differentiated cells (3d diff and 6d diff) were infected with WNV and DENV-2 virus at an MOI of 1 or 5, and viral titers were assessed at times 0, 24, 48, and 72 h. Vero cells were maintained as positive controls for infection and virus replication. In the WNV-infected cells, a similar trend to VEEV TC-83 was observed, where there was a significant decrease in viral titer in 6d differentiated LUHMES cells compared to 3d differentiated LUHMES cells at an MOI of 1. In cells infected with DENV-2 at an MOI of 5, there was a decrease in 3d and 6d differentiated LUHMES cells compared to undifferentiated and Vero cells, although viral titers between 3d and 6d were comparable. In contrast, in cells infected with DENV-2 at an MOI of 5, the trend of reduced viral titers as the differentiation period increased was maintained until the 72 hpi time point.

Viral infectivity of the differentiated cells was further verified by immunostaining for VEEV E2, a structural protein marker. Hoechst staining was included as a nuclear marker, and phalloidin (filamentous actin marker) was used in undifferentiated and 3d and 6d differentiated LUHMES cells infected with VEEV TC-83 at an MOI of 0.1 at 24 hpi (Figure 2B). As expected, consistent Hoechst staining (nuclear) was observed regardless of differentiation, while there was a decrease in VEEV E2 expression as the period of differentiation increased. These data supported plaque assay data that, similar to mature neurons, differentiated LUHMES cells are less permissive than undifferentiated LUHMES cells. Assessment of the phalloidin stain (filamentous actin) accentuated the structural differences between undifferentiated and differentiated LUHMES, where there is a diffusion of phalloidin observed from the neuronal cell body towards the axon terminal in differentiated LUHMES and an aggregation of phalloidin in undifferentiated LUHMES cells.

**Figure 3:**
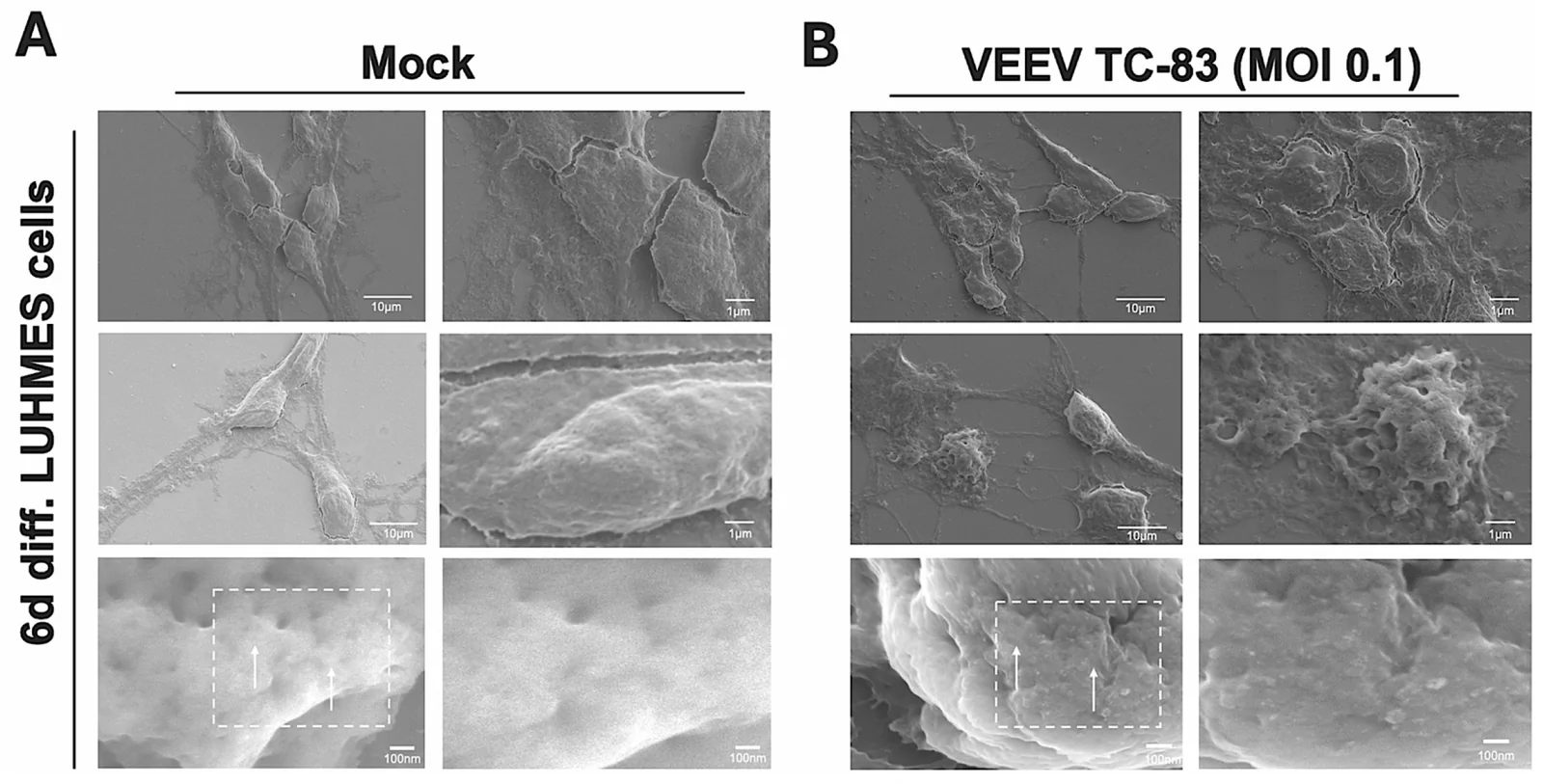
Assessment of morphological changes in 6-day differentiated LUHMES cells infected with VEEV TC-83 by SEM. Representative SEM images of VEEV TC-83-infected (0.1 MOI) and uninfected 6-day differentiated (6d diff) LUHMES cells at 16 hpi. (**A**) Uninfected mock images showing cell axon connectivity and surface morphological integrity of the cell body, with magnified regions of the cell body shown in the bottom images. (**B**) Infected images showing cell axon connectivity and surface morphological integrity of the cell body, with magnified regions of the cell body in the bottom images.

Morphological changes in 6-day differentiated LUHMES cells following 16 h infection with VEEV TC-83 (MOI 0.1) were evaluated by scanning electron microscopy (SEM) (Figure 3 and Figure S3A). This study revealed subtle changes in the connections between the cell body and axons, with some extent of loss of intercellular connections noted in the infected cells [35]. Representative images in Figure 3 show changes in the surface integrity of the cell body that appear to be more ragged in infected LUHMES cells, in contrast to the smooth and uninterrupted cell body of uninfected (mock) cells. Furthermore, in infected cells, as indicated with white arrows, there appears to be slight surface blebbing from the highlighted dashed box area. Overall, SEM studies demonstrated that infected LUHMES cells undergo loss of intercellular connectivity and display morphological changes on the surface of the neuronal cell body. A quantitative morphometric analysis and standardized scoring are required to understand these data further.

### 3.3. Efficacy Assessment of a Small Molecule Inhibitor, Omaveloxolone, in Differentiated LUHMES Cells

**Figure 4:**
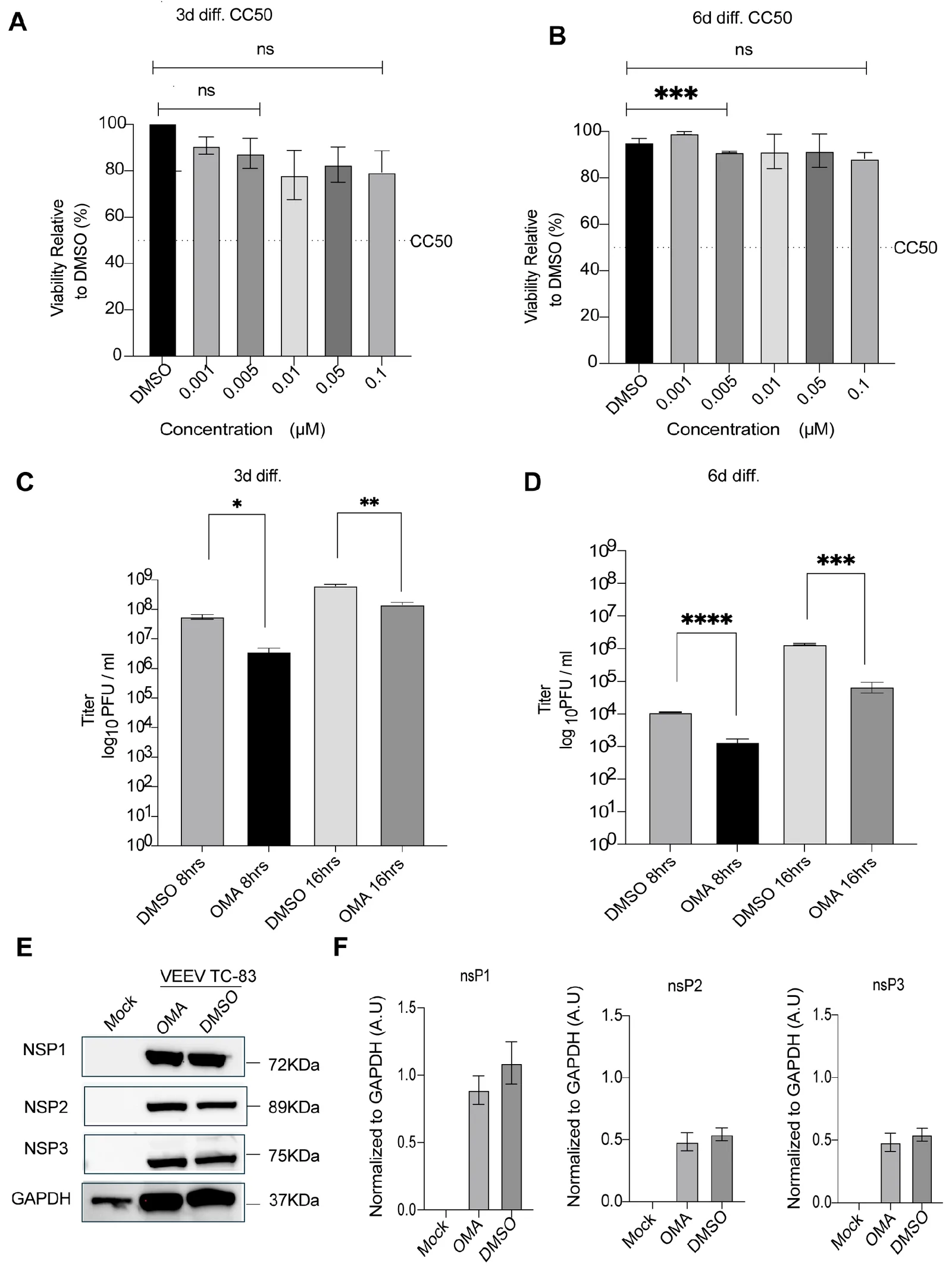
Cytotoxicity of Omaveloxolone (OMA) and treatment impact on VEEV TC-83 replication and protein expression in 3d and 6d differentiated LUHMES cells. LUHMES cells were differentiated for 3-days (3d diff) or 6 days (6d diff). Cytotoxicity of OMA was assessed at various concentrations in 3d (**A**) and 6d (**B**) differentiated cells. Efficacy of treatment (either OMA or DMSO) in VEEV TC-83-infected (MOI 0.1) LUHMES, either 3d (**C**) or 6d (**D**) differentiated, was also assessed at 8 and 16 h post-infection (hpi). (**E**) Protein expression of VEEV nsP1, nsP2, nsP3, and GAPDH as a loading control was assessed in 6d differentiated LUHMES cells at 16 hpi using two independent runs. (**F**) Protein expression was normalized to GAPDH (n = 2 independent experiments). Statistics for (**C**,**D**) were done using an unpaired, two-tailed *t*-test and one-way ANOVA for A and B compared to appropriate controls. Values are the average of three biological replicates ± standard deviation. Individual time points were analyzed via one-way ANOVA and *t*-test; * *p* < 0.05, ** *p* < 0.01, *** *p* < 0.001, **** *p* < 0.0001, *p* = ns (not significant).

**Figure 5:**
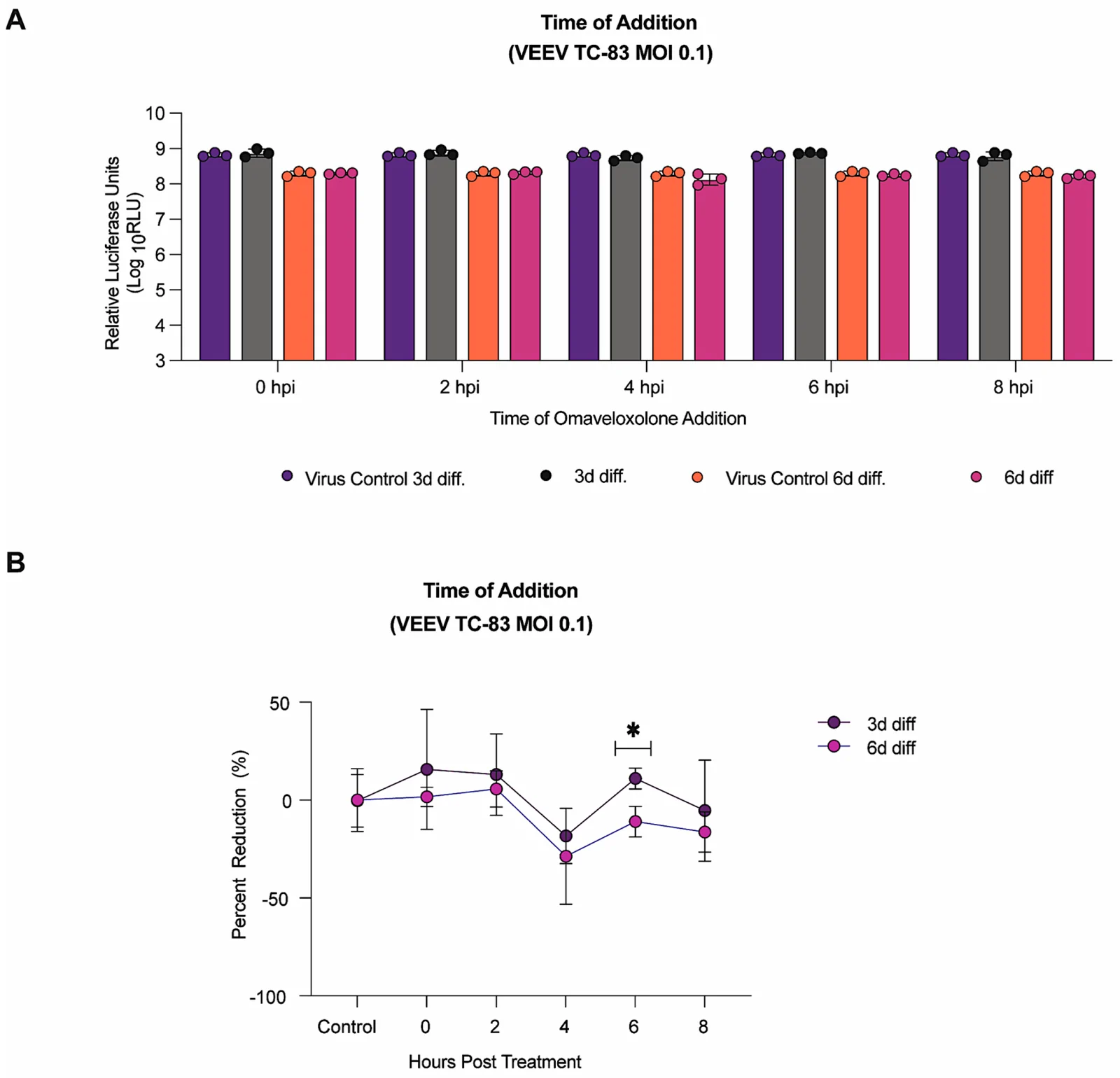
Time-of-addition assay for OMA-mediated inhibition in 3d and 6d differentiated LUHMES cells. Time-of-addition assay evaluating the antiviral activity of OMA at 0.001 μM against VEEV TC-83 luciferase-tagged virus (MOI = 0.1). OMA was added at 0, 2, 4, 6, or 8 h post-infection (hpi), and viral replication was assessed by luciferase reporter activity in 3d and 6d differentiated LUHMES cells. (**A**) Relative luciferase activity was normalized to the untreated-cell control for virus-only infected cells and to the OMA-only treated control for OMA-treated infected cells to account for background luminescence. (**B**) Percent reduction in viral replication following OMA treatment at the indicated times of post-infection. Percent reduction was calculated relative to the corresponding virus-infected control (Control) for each differentiation time point (3d diff. and 6d diff.). Analyses were based on three replicates, each using *t*-tests; * *p* < 0.05.

To investigate whether differentiated LUHMES cells would be responsive to treatment, we utilized Omaveloxolone (OMA), an activator of transcription factor Nuclear factor erythroid 2-related factor 2 (Nrf2) and an inhibitor of the Nuclear Factor kappa-light-chain-enhancer of activated B cells (NF-κB) pathway, which has previously been shown to exert a robust inhibition of neurotropic Alphaviruses in multiple CNS-relevant cell types [36,37]. Firstly, cytotoxicity was assessed in 3-day and 6-day differentiated LUHMES cells at concentrations ranging from 0.001 to 0.1 µM for 24 h. This study revealed a dose-dependent reduction in viability in 3-day differentiated LUHMES cells, with average cell viability ranging between 78 and 90% (Figure 4A). However, 6-day differentiated LUHMES cells were more tolerant to OMA treatment, with cell viability ranging between 88 and 99% at all concentrations tested (Figure 4B). Based on these results, a concentration of 0.001 µM, where cell viability was >90% in both 3d and 6d differentiated LUHMES cells, was selected for subsequent experiments. Next, infection studies with VEEV TC-83 (MOI 0.1) were conducted in 3d and 6d differentiated LUHMES cells following pre- and post-treatment with OMA. Viral titer was assessed at 8 and 16 hpi. Both OMA-treated 3d and 6d differentiated LUHMES cells resulted in significant decreases in viral titer compared to DMSO controls at both time points (Figure 4C,D). The 3d differentiated LUHMES cells resulted in a 15-fold and 4-fold reduction in viral titer at 8 and 16 hpi, respectively. The 6d differentiated LUHMES cells resulted in an 8-fold and 20-fold reduction in viral titer at 8 and 16 hpi, respectively. Lysates from 6d differentiated LUHMES cells pre- and post-treated with OMA (0.001µM) and infected with VEEV TC-83 (MOI 0.1) at 16 hpi were used to evaluate protein expression of viral nonstructural proteins, nsP1, nsP2, and nsP3 (S3B). Western blots probed for nsP1–3 and re-probed for GAPDH for normalization revealed no significant differences in viral protein expression in OMA-treated cells compared to DMSO-treated cells Figure 4E,F and Figure S3C). A time-of-addition study (Figure 5) of OMA (0.001 µM) at 0, 2, 4, 6, and 8 hpi was assessed in 3d and 6d differentiated LUHMES cells with luciferase-tagged VEEV TC-83. Overall, there was no significant decrease in luciferase activity when compared to their respective controls. Importantly, OMA-mediated antiviral inhibition was sustained even when treatment was initiated 4 h post-infection. Overall, these studies showed that differentiated LUHMES cells are responsive to host-directed therapeutics, such as OMA, with 6d differentiated cells exerting a better response by increased cell viability and decreased viral titer.

These results were further expanded utilizing our two representative Flaviviruses, WNV and DENV-2 (Figure S4). Viral titer was assessed at 24 and 48 hpi for WNV-infected cells, while 48 and 72 hpi were evaluated for DENV-2-infected cells at an MOI of 5. Focused on 48 hpi, there was a 7-fold (3d) and 27-fold reduction in viral titer as compared to DMSO control in DENV-2-infected cells. In WNV infected cells, there was an 8472-fold (3d) and 464-fold (6d) reduction in viral titer when infected with WNV.

### 3.4. Investigation of Transcriptional Changes and Phospho-Signaling Events in LUHMES Cells Following Infection with VEEV TC-83 and Treatment with OMA

To obtain proof-of-concept data pertaining to the host cell response of LUHMES cells to infection with and without treatment with OMA, we analyzed neurotoxicity responses by gene expression analysis and phospho-signaling events by Reverse Phase Protein Array (RPPA) proteomic analysis.

**Figure 6:**
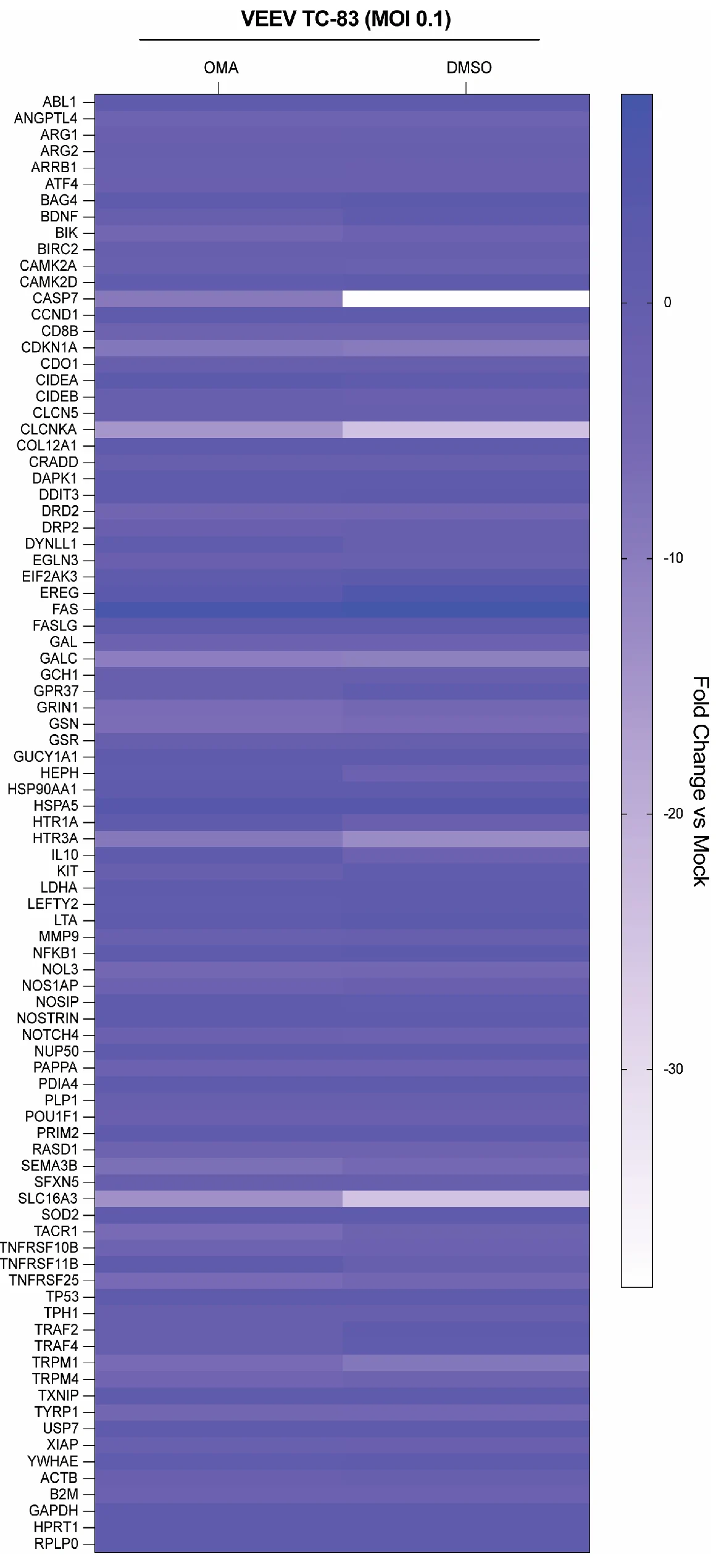
Heatmap of neurotoxicity-related genes impacted by VEEV TC-83 infection in DMSO- or OMA-treated, 6-day differentiated LUHMES cells. The 6-day differentiated LUHMES cells were either OMA- or DMSO-treated and infected with VEEV TC-83 at an MOI of 0.1. RNA was extracted at 16 h post-infection (hpi). Expression of neurotoxicity-related genes was assessed via a gene expression array (Neurotoxicity RT^2^ Profiler). Color change indicates fold increase or decrease relative to the untreated infected control (mock) (n = 4).

Analysis of transcription activity of neurotoxicity-related genes was conducted using 6d differentiated LUHMES cells, which were either infected with VEEV TC-83 for 16 h and treated with DMSO, infected and treated with 0.001 µM of OMA, or uninfected and untreated mock controls. The relative gene expression patterns of treatment groups compared to the mock control group are shown as a heat map (Figure 6) and significant fold change values in a table (Table 3). Before conducting neurotoxicity analysis, viral titer was verified by plaque assay and qRT-PCR as a quality control step (Figure S5). In our analysis, gene expression was normalized to the mock-infected control, and the reported fold changes represent the response of each treatment group relative to the mock control group. CASP7 expression was decreased by −38.52-fold in VEEV-infected DMSO-treated cells and by −9.22-fold in VEEV-infected OMA-treated cells relative to mock, indicating that the suppression of CASP7 expression was less pronounced in the OMA-treated group. In contrast, GRIN1 expression remained comparably downregulated in both infected groups relative to the mock control group (−5.07-fold in DMSO-treated cells and −6.45-fold in OMA-treated cells), suggesting that OMA did not substantially restore expression of this synaptic gene.

We therefore interpret these findings as evidence that OMA does not uniformly reverse virus-induced transcriptional changes. Rather, its effects appear to be gene-specific, with some genes showing attenuation of virus-induced dysregulation while others remain largely unaffected (Table 3). Furthermore, there was a pronounced downregulation of neuronal and synaptic signaling genes, including *HTR3A*, *TACR1*, *TRPM1*, *TRPM4*, *CAMK2A*, and *NOS1AP*, consistent with impaired glutamatergic transmission, calcium signaling, and overall neuronal function in DMSO-treated cells. Ion transport and metabolic regulators such as *CLCNKA* and *SLC16A3* were also strongly suppressed. Additionally, key apoptotic and cell death-related genes, including *CASP7*, *CDKN1A*, *TNFRSF25*, *TNFRSF10B*, *BIK*, and *NOL3*, were downregulated. The 6d differentiated LUHMES cells that were OMA-treated partially abrogated the downregulation of several neurotoxicity-associated genes, particularly *CASP7*, *SLC16A3*, *CLCNKA*, *HTR3A*, and *TACR1*, suggesting a moderating effect on infection-induced neuronal suppression. Although some stress-response genes, such as *DDIT3*, *CIDEA*, and *EREG*, remained upregulated in both conditions, the data indicate that OMA treatment mitigates, but does not completely reverse, VEEV-induced transcriptional changes associated with neuronal dysfunction and apoptotic signaling. Upregulation of FAS by infection is 8.16 in DMSO-treated cells compared to the 7.22 upregulation in OMA-treated cells at 0.001 µM. This shows partial rescue of apoptotic events caused by infection. These data emphasize the need to further investigate dosing mechanisms and molecular drivers of neurotoxicity following an infection with encephalitic RNA viruses.

**Figure 7:**
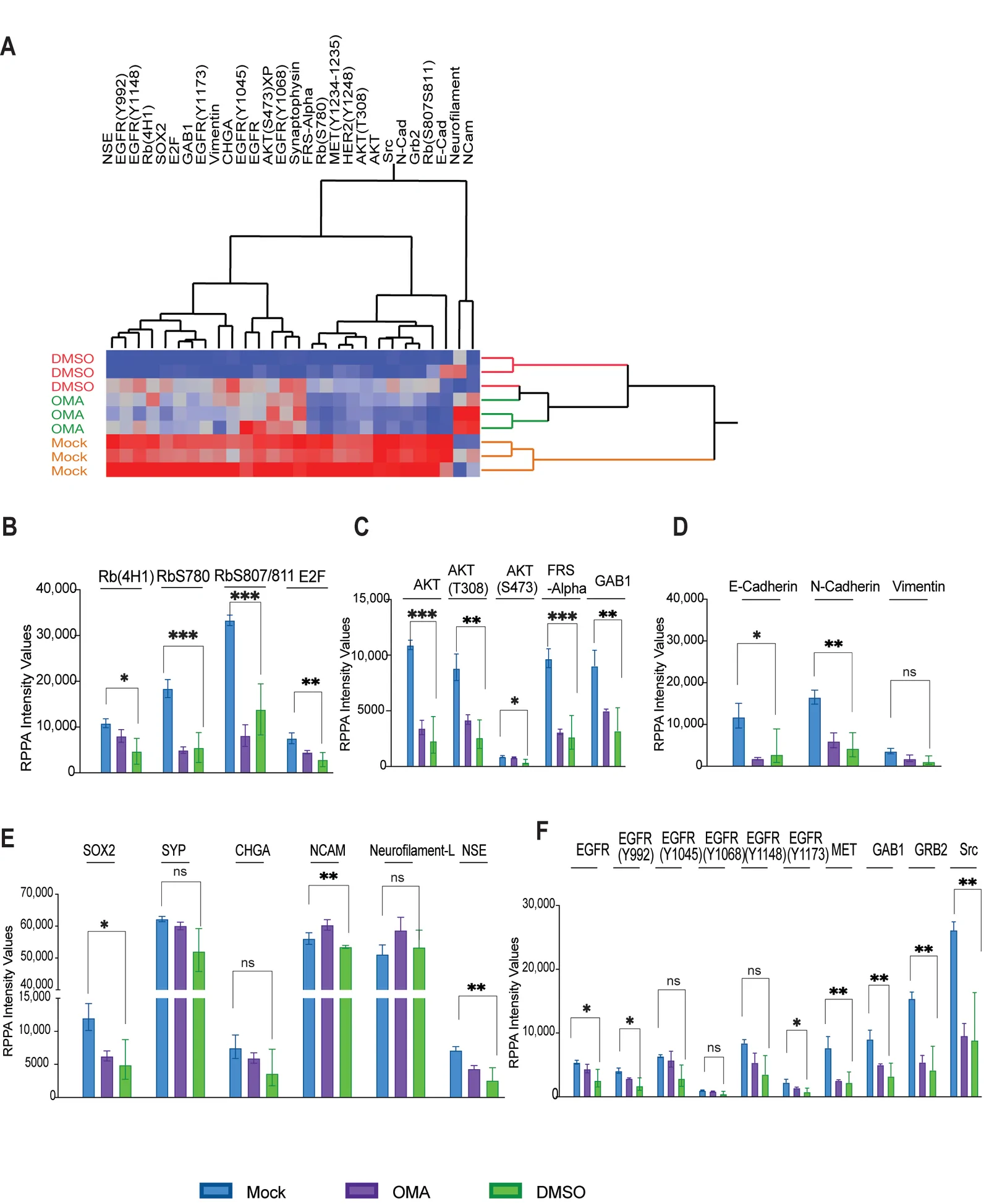
Analysis of intracellular phospho-signaling pathways in VEEV TC-83-infected LUHMES cells, either DMSO-treated or OMA-treated. (**A**) Dendrogram of the averaged samples of Mock, infected DMSO-treated, and infected OMA-treated cells. (**B**) Cell cycle pathway proteins of Mock, infected DMSO-treated, and infected OMA-treated cells. (**C**) AKT signaling pathway proteins of Mock, infected DMSO-treated, and infected OMA-treated cells. (**D**) Cell lineage proteins of Mock, infected DMSO-treated, and infected OMA-treated cells. (**E**) Neuronal lineage proteins of Mock, infected DMSO-treated, and infected OMA-treated cells. (**F**) RTK signaling proteins of Mock, infected DMSO-treated, and infected OMA-treated cells. Statistical analysis was based on the average of three biological replicates, * *p* < 0.05, ** *p* < 0.01, *** *p* < 0.001, and *p* = ns (not significant).

Proteomic inquiry of host intracellular signaling events that are impacted by infection and drug treatment was conducted by reverse-phase protein arrays (RPPA) as a proof-of-concept analysis of proteomic alterations occurring in LUHMES cells focused on phospho-signaling events. Signaling pathways focused on neuroplasticity were evaluated as a prototypical signaling target. The following conditions were assessed: (1) Mock control group (uninfected, untreated), (2) DMSO (infected, DMSO-treated), and (3) OMA (infected, OMA-treated at 0.001 µM). Prior to RPPA analysis, viral titer was verified by plaque assay as a quality control step for each condition (Figure S7A). The results of the RPPA analyses were averaged and represented as a dendrogram with hierarchical clustering similarities observed amongst the different conditions (Figure 7A and S7B). As expected, clear phospho-signaling profiles were observed between each group. The dendrogram also shows distinct changes in the treated groups (DMSO/OMA) as compared to the mock control group.

OMA treatment induced changes in cellular adhesion and EMT-associated signaling events (Figure 7B). E-cadherin, a classical epithelial protein required for stable cell–cell junctions, was reduced in the OMA-treated cells relative to the mock control group (Figure 7D). In contrast, N-cadherin was elevated with OMA treatment when compared to mock control cells. Vimentin, a canonical mesenchymal marker, remained higher in the DMSO-treated cells, unlike the OMA-treated cells. Together, these differences suggest that OMA treatment of infected wells partially restored adhesion marker expression. SOX2 signaling appeared lower in OMA-treated, infected cells when compared to mock cells, suggesting OMA treatment caused transcriptional reprogramming in the treated cells (Figure 7E). This confirms OMA’s mechanism as an *NRF2* activator, which upregulated the expression of Nrf2. OMA treatment also influenced neuroendocrine signaling in the infected, DMSO-treated cells. Synaptophysin and neurofilament-L were elevated in OMA-treated, infected cells relative to DMSO-treated, infected cells. Chromogranin A and NCAM/CD56 showed a similar pattern, indicating OMA treatment shifted cells towards a neuroendocrine-like state more similar to mock control cells. Considering receptor tyrosine kinase signaling (RTK), multiple phosphorylation sites of EGFR were altered, including Y1045, Y1068, Y1148, Y1173, and Y992 (Figure 7F). These phosphorylation sites regulate receptor activation and internalization. Additionally, EGFR and ErbB2 were also differentially expressed. AKT (T308 and S473) and Src were modulated by treatment, showing lower relative expression in OMA-treated, infected cells as compared to DMSO-treated, infected cells, more comparable to mock cells (Figure 7C). Taken together, this clustering analysis reveals that OMA-treated, infected cells resembled signaling interactions of mock control cells, showing partial neuronal rescue with OMA treatment. OMA displayed a clustering profile towards mock cells and showed partial rescue of activation of survival pathways. A validation experiment (S6) was performed to include DMSO-only and OMA-only treatment control groups on selected candidate genes (CASP7 and EREG) (Figure S6A,B) to be independently analyzed by RT-qPCR, while changes in E2F and SOX2 protein expression (Figure S6C,D) were evaluated by Western blotting to distinguish the effects of the vehicle controls from those of OMA treatment. Consistent with the PCR array results, CASP7 expression was reduced following treatment, whereas EREG expression showed no significant differences among the treatment control groups or infected groups. Western blot analysis further demonstrated treatment-associated changes in E2F and SOX2 protein abundance in the presence of infection versus control groups, while providing additional support that the observed molecular changes are attributable to treatment rather than vehicle effects. Further investigation is required to capture the granularity of the observations from this study.

## 4. Discussion

Arthropod-borne viruses infect hundreds of millions of people globally every year. While some infections result in mild and asymptomatic disease, a subset of infections, such as those caused by the Alphavirus and Orthoflavivirus genera, are neurotropic and cause CNS-related disease [38,39]. Affected individuals often face limited or no treatment options, with lasting neurological damage in the form of neurological sequelae that impact their quality of life long-term. The neurological manifestations of infection-induced disease are a multicellular phenomenon involving different CNS cell types, with the prominent cells being neurons. Inflammation induced by glial cells adds to the neuronal pathology [40,41]. There is a lack of clinically relevant, simple, and affordable in vitro models that can recapitulate salient features of human disease and assess preclinical therapeutic pipelines to address short- and long-term neurological disease caused by these viral infections.

Previous research has used the SH-SY5Y cell line to study virus-induced neurological diseases, such as HSV-1, measles, and rabies viral infections [14,28]. However, this immortalized neuroblastoma cell line, originating from the bone marrow of a cancer patient, expresses cancer-like characteristics and genetic alterations that may not align with the acute pathology caused by these infections. Rodent-derived neurons lack essential physiological properties relevant to studying human disease from these viruses [42,43]. Human-induced pluripotent stem cell (hiPSC)-derived neural models have emerged as valuable tools for studying both infectious and noninfectious neurological diseases. hiPSC-derived neurons, astrocytes, microglia, and brain organoids have been widely used to investigate neurodevelopmental disorders, neurodegenerative diseases, and viral infections, including Zika virus, West Nile virus, and Japanese encephalitis virus [44,45,46]. However, hiPSC-based platforms possess some limitations, including lengthy differentiation timelines, high cost, batch-to-batch variability, technical complexity, and the persistence of fetal-like transcriptional signatures that may not fully represent mature adult neurons [47]. These challenges can limit scalability and reproducibility for high-throughput antiviral screening applications.

To address these limitations, the Lund Human Mesencephalic (LUHMES) neuronal precursor cell line can be considered an attractive alternative human neuronal model [20,22]. LUHMES cells can be rapidly differentiated into mature post-mitotic dopaminergic-like neurons within days, exhibit robust neuronal morphology and function, and demonstrate high experimental reproducibility [17,21,48,49].

The data presented in this study demonstrate a scalable, validated method to generate differentiated LUHMES cells, recapitulating physiological aspects of mature human neuronal cells. Our data show that differentiated LUHMES cells are susceptible yet less permissive than undifferentiated LUHMES cells to VEEV TC-83, WNV, and DENV-2. This finding is supported by previous studies where mature CSM14.1 rat neurons were less permissive than immature neurons to Sindbis virus (SINV) [33,50]. The morphological analyses presented here further reinforce the relevance of LUHMES cells as a model for studying neurons. SEM imaging revealed that VEEV infection disrupts neuronal connectivity and alters cell surface integrity, producing phenotypes reminiscent of neurodegenerative processes. The observed loss of neurite connections and increased membrane blebbing are consistent with cellular stress responses, cytoskeletal destabilization, and early apoptotic events [35,51]. The SEM approach is limited to the assessment of extracellular and surface features. Therefore, future studies will need to incorporate transmission electron microscopy (TEM) to characterize intracellular ultrastructural changes associated with infection, including viral replication sites, organelle alterations such as those of mitochondria, membrane rearrangements, and cytopathic effects [52,53]. Furthermore, we showed how OMA treatment partially recovered critical antiviral host responses in LUHMES cells in the presence of infection, as shown in previous studies [36,37]. These findings are corroborated by earlier publications, which showed that Nuclear Factor kappa-light-chain-enhancer of activated B cells (NF-κB) activation promotes Alphavirus replication in mature neurons [54,55]. The neurotoxicity analyses provide deeper insight into the molecular mechanisms underlying infection-induced neuronal dysfunction. Notable genes such as GRIN1 and CAMK2A, which are essential for synaptic plasticity and neurotransmission, were significantly suppressed (Table 3). This transcriptional repression likely contributes to impaired neuronal communication and may contribute to cognitive and motor deficits associated with neurotropic viral infections [49,56]. The phospho-signaling data further complement these findings by revealing alterations in key signaling pathways associated with cell survival, proliferation, and neuronal identity. Infection-induced changes in AKT, Src, and receptor tyrosine kinase signaling pathways reflect a shift toward a dysregulated cellular state that may favor viral replication or contribute to cell death [57,58,59].The restoration of neuroendocrine markers such as synaptophysin and neurofilament-L following OMA treatment further supports the notion that host-directed therapies can promote recovery of neuronal characteristics. While the data presented suggest OMA treatment resulted in a neuroprotective but non-physiological state in LUHMES, further studies are needed to distinguish between rescue and pharmacological reprogramming.

This study is presented as a foundation for studying neurotropic viral infections in a human context; however, the study has some limitations. The viruses included in this study are representative viruses from their genus; however, adding more isolates from different subgroups, including the wild-type VEEV strain (VEEV IAB) from which VEEV TC-83 was derived, would strengthen the observed differences between VEEV TC-83, WNV, and DENV-2. Further studies are needed to dissect the host mechanisms impacted by OMA treatment in DENV-2-infected cells. The differentiation-associated remodeling of the transcriptome and proteome, including the reduced expression of proliferation-associated factors and increased expression of neuron-specific proteins specific to LUHMES cells, needs to be investigated further to get a more complete picture of the transcriptional reprogramming that is induced by the infection and to appreciate the proteomic signaling alterations. Such expanded transcriptomic and proteomic studies will also help in the identification of more host-based therapeutic targets. Taken together, the proof-of-concept data presented in this study provide a comprehensive foundational understanding of the application value of LUHMES cells to study how neurotropic viruses interact with human neurons and how these interactions can be modulated by therapeutic intervention.

## Figures and Tables

**Table 3:** Assessment of selected neurotoxicity-related genes impacted by Omaveloxolone (OMA) or DMSO treatment following infection with VEEV TC-83. The 6-day differentiated LUHMES cells were either OMA- or DMSO-treated and infected with VEEV TC-83 at an MOI of 0.1. RNA was extracted at 16 h post-infection (hpi). Expression of neurotoxicity-related genes was assessed via a gene expression array (Neurotoxicity RT^2^ Profiler). Values represent fold-increase or decrease relative to the untreated infected control, with corresponding *p*-values.

Genes	OMA	*p* Value	DMSO	*p* Value
*CLCNKA*	−15.39	0.02437	−24.64	0.02385
*SLC16A3*	−13.97	0.00696	−24.89	0.00054
*CASP7*	−9.22	0.02543	−38.52	0.02579
*HTR3A*	−9.08	0.00022	−13.1	0.00017
*CDKN1A*	−8.69	1.4 × 10^−5^	−9.53	1.1 × 10^−5^
*SEMA3B*	−7.07	0.00356	−5.43	0.00428
*GRIN1*	−6.45	0.00201	−5.07	0.0026
*TNFRSF25*	−6.23	0.00487	−4.97	0.00596
*TACR1*	−6.15	8.6 × 10^−5^	−3.78	0.00097
*TRPM1*	−6.05	0.0285	−8.69	0.0295
*NOL3*	−5.03	0.00016	−4.94	0.00015
*BIK*	−4.92	0.0037	−2.97	0.00759
*TRPM4*	−4.29	0.00078	−3.25	0.00134
*TNFRSF10B*	−3.05	0.00069	−2.64	0.00034
*NOS1AP*	−2.42	0.00697	−1.67	0.02483
*CAMK2A*	−1.9	0.00143	−2.13	0.00329
*DDIT3*	1.93	0.0369	2.17	0.00108
*CIDEA*	2.27	0.30612	1.23	0.5809
*EREG*	2.86	0.17695	5.94	0.07226
*FAS*	7.22	0.0864	8.16	0.03675
Upregulated by OMA and DMSO				
Increased downregulation by OMA				
Decreased downregulation by OMA				

Grey: Upregulated by OMA and DMSO; white: Increased downregulation by OMA; green: Decreased downregulation by OMA.

## Data Availability

The original contributions presented in this study are included in the article/Supplementary Materials. Further inquiries can be directed to the corresponding authors.

## Supplementary Materials

The following supporting information can be downloaded at: https://www.mdpi.com/article/10.3390/microorganisms14091944/s1, Figure S1: Validation of β-III tubulin neuronal marker expression; Figure S2: Susceptibility and replication kinetics of West Nile virus (WNV) and Dengue virus serotype 2 (DENV-2) in both Vero cells and undifferentiated/differentiated LUHMES cells; Figure S3: Infectious virus titers and whole-blot images used for scanning electron microscopy (SEM) and western blot analyses; Figure S4: Impact of Omaveloxolone treatment on WNV and DENV-2 replication in 3d and 6d differentiated LUHMES cells; Figure S5: Quantification of viral RNA via qRT-PCR of VEEV TC-83-infected LUHMES cells for gene expression analysis; Figure S6: Neurotoxicity and phospho-signaling validation of independent genes and protein expression; Figure S7: Infectious virus titers and hierarchical clustering analysis from RPPA study; Table S1: Antibodies for RPPA.
