# Supplementary material for: Differentiated LUHMES Cells as a Model to Investigate Neurotropic Arboviruses and Evaluate Host-Directed Therapeutics
Source: Microorganisms. 2026 Sep 2;14(9):1944. doi: 10.3390/microorganisms14091944 (PMC13609019; doi:10.3390/microorganisms14091944)
Supplement: Supplementary file 1 [file microorganisms-14-01944-s001.zip › microorganisms-4409884-supplementary.pdf]

Supplementary Figures and Tables

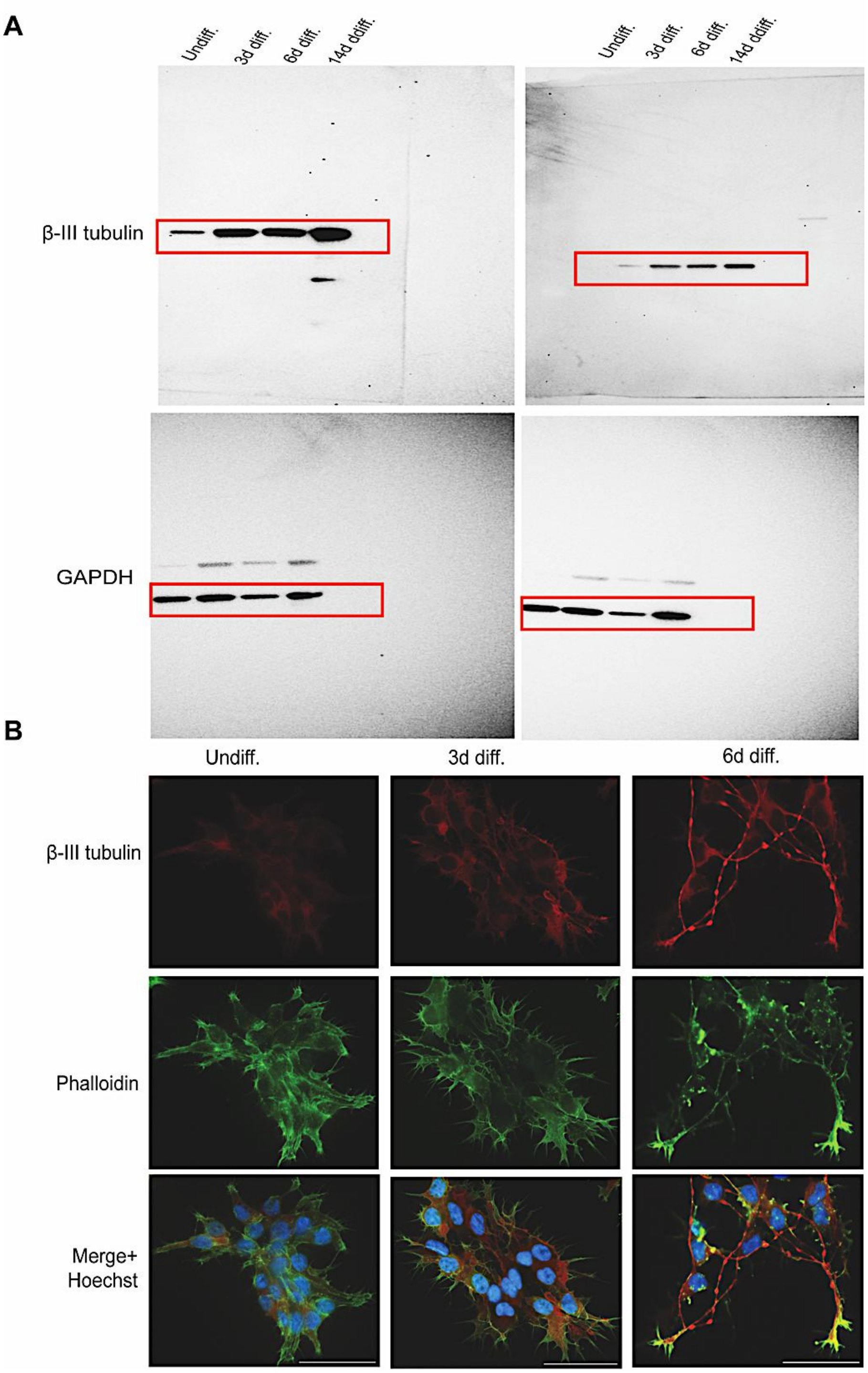

Supplemental Figure S1. Validation of β-III tubulin neuronal marker expression. (**A**) Representative whole-blot images evaluating the neuronal marker, β-III tubulin, and loading control, GAPDH, in undifferentiated and 3d/6d/14d differentiated LUHMES cells. (**B**) 60x IF images of Undiff., 3d diff. and 6d diff. β-III tubulin staining. Scale bars are 10 μm.

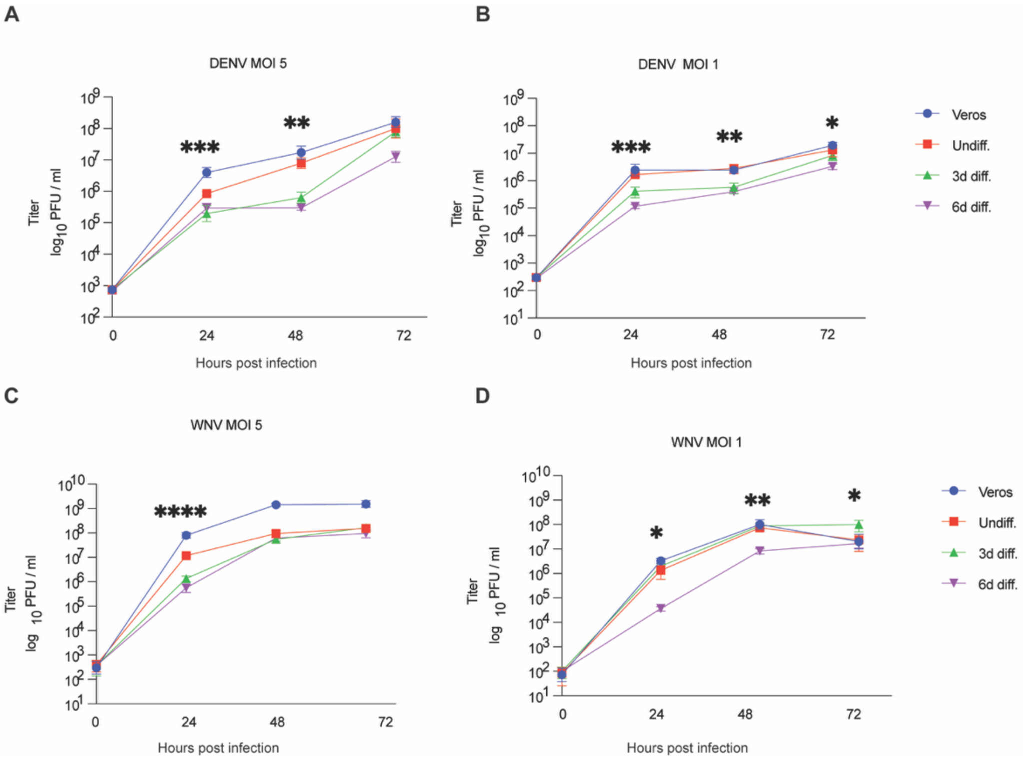

Supplemental Figure S2. Susceptibility and replication kinetics of West Nile virus (WNV) and Dengue virus serotype 2 (DENV-2) in both Vero cells and undifferentiated/differentiated LUHMES cells. (**A**) Plaque viral titer quantification of DENV-2 at MOI of 5 and MOI of 1. (**B**) Plaque viral titer quantification of WNV at MOI of 5 and MOI of 1. Values are the average of three biological replicates ± standard deviation. Individual time points were analyzed via one-way ANOVA relative to undifferentiated cells; **$p < 0.05$, **$p < 0.01$, ***$p < 0.001$, ****$p < 0.0001$,* nonsignificant have time points have no p-value.

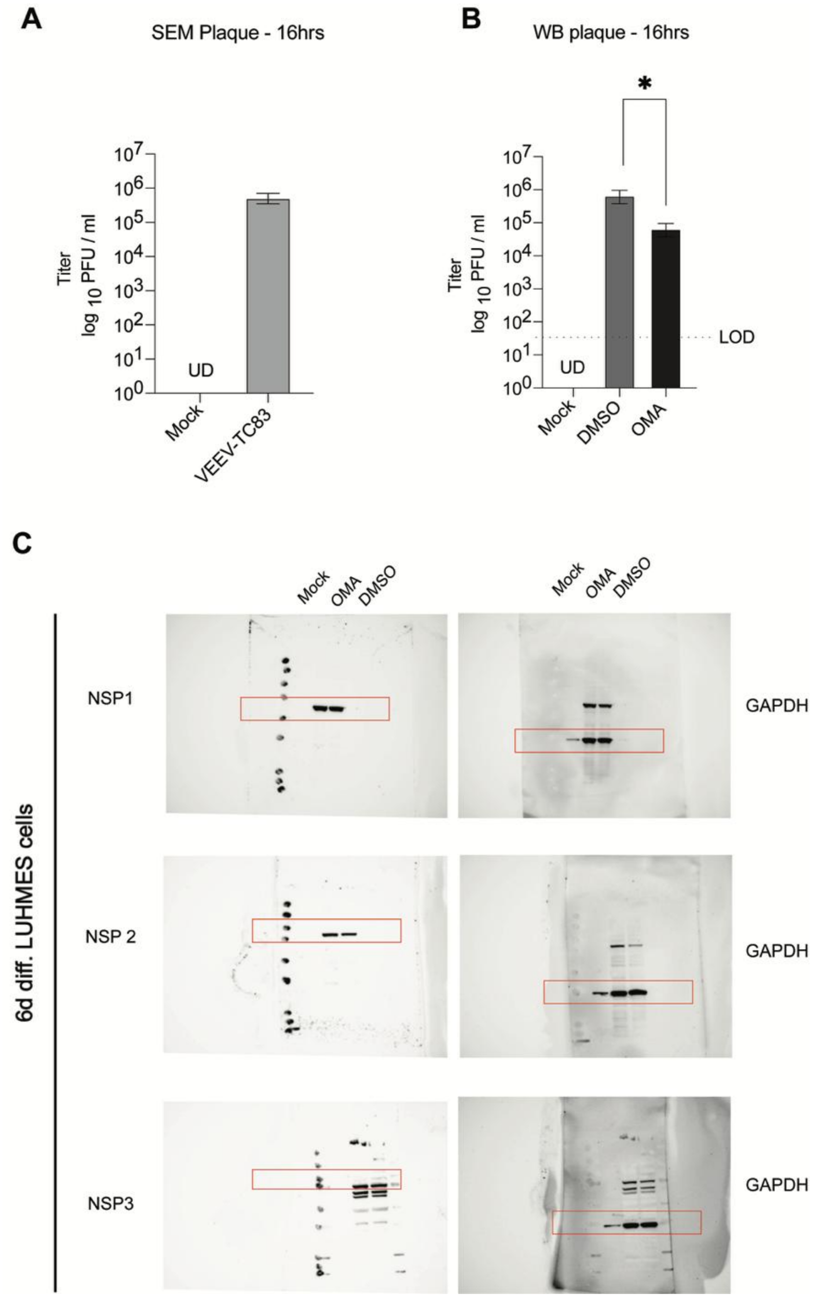

Supplemental Figure S3.
Infectious virus titers and whole-blot images used for scanning electron microscopy (SEM) and western blot analyses. **(A)** Infectious virus titers in culture supernatants collected at 16 hours post-infection (hpi) from samples used for SEM analysis, quantified by plaque assay following infection at a multiplicity of infection (MOI) of 0.1. **(B)** Infectious virus titers in culture supernatants from samples used for western blot analysis, quantified by plaque assay at 16 hpi following infection at an MOI of 0.1. **(C)** Uncropped western blot images showing expression of the VEEV nonstructural proteins NSP1, NSP2, and NSP3 in 6-day differentiated LUHMES cells infected at an MOI of 0.1 and harvested at 16 hpi. Significance is the average of three biological replicates using a t-test analysis; $*p < 0.05$.

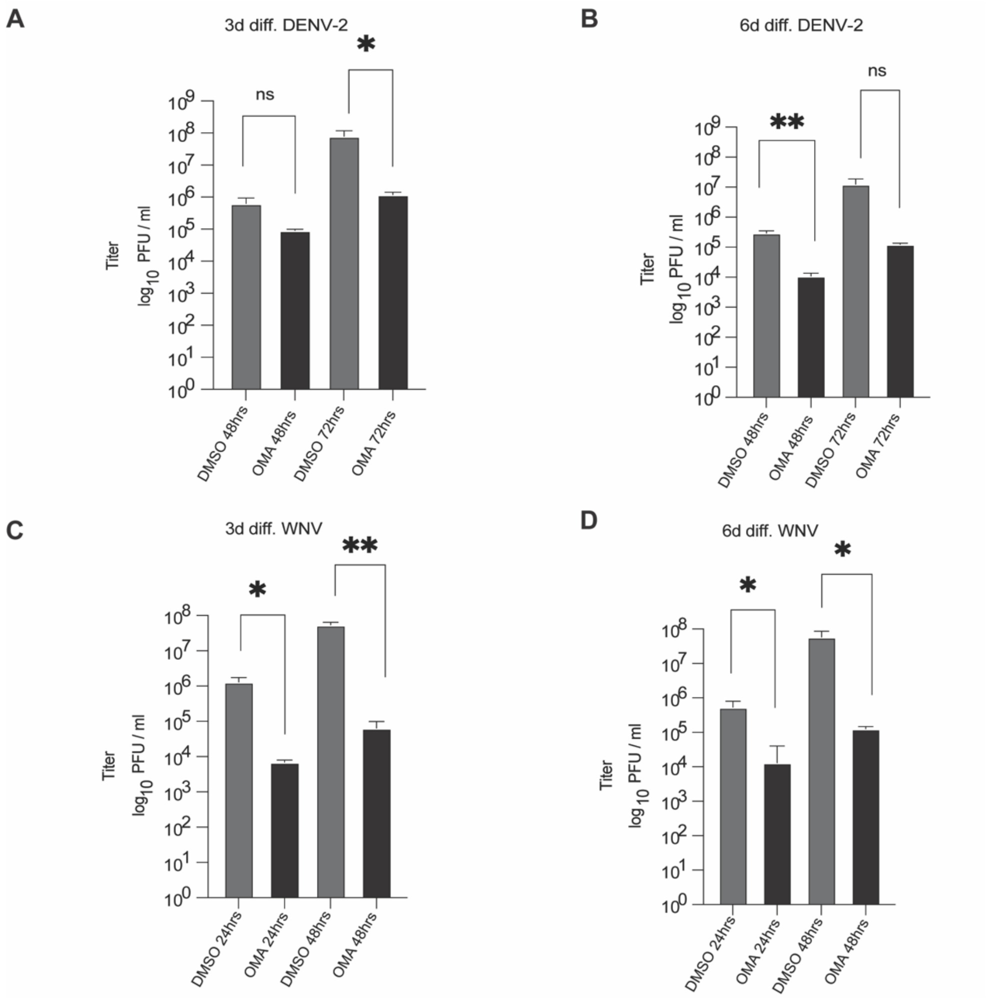

Supplemental Figure S4. Impact of Omaveloxolone treatment on WNV and DENV-2 replication in 3d and 6d differentiated LUHMES cells at an MOI of 5. Plaque quantification of viral titers assessing the efficacy of OMA treatment in 3d diff (**A**) and 6d diff (**B**) infected DENV-2 cells. Plaque quantification of viral titers assessing the efficacy of OMA treatment in 3d diff (**C**) and 6d diff (**D**) of WNV-infected cells. Statistical analysis used an unpaired t-test analysis: *$p < 0.05$*, ** *$p < 0.01$*, and *$p = ns$* (not significant). Statistical analysis was based on three technical replicates each.

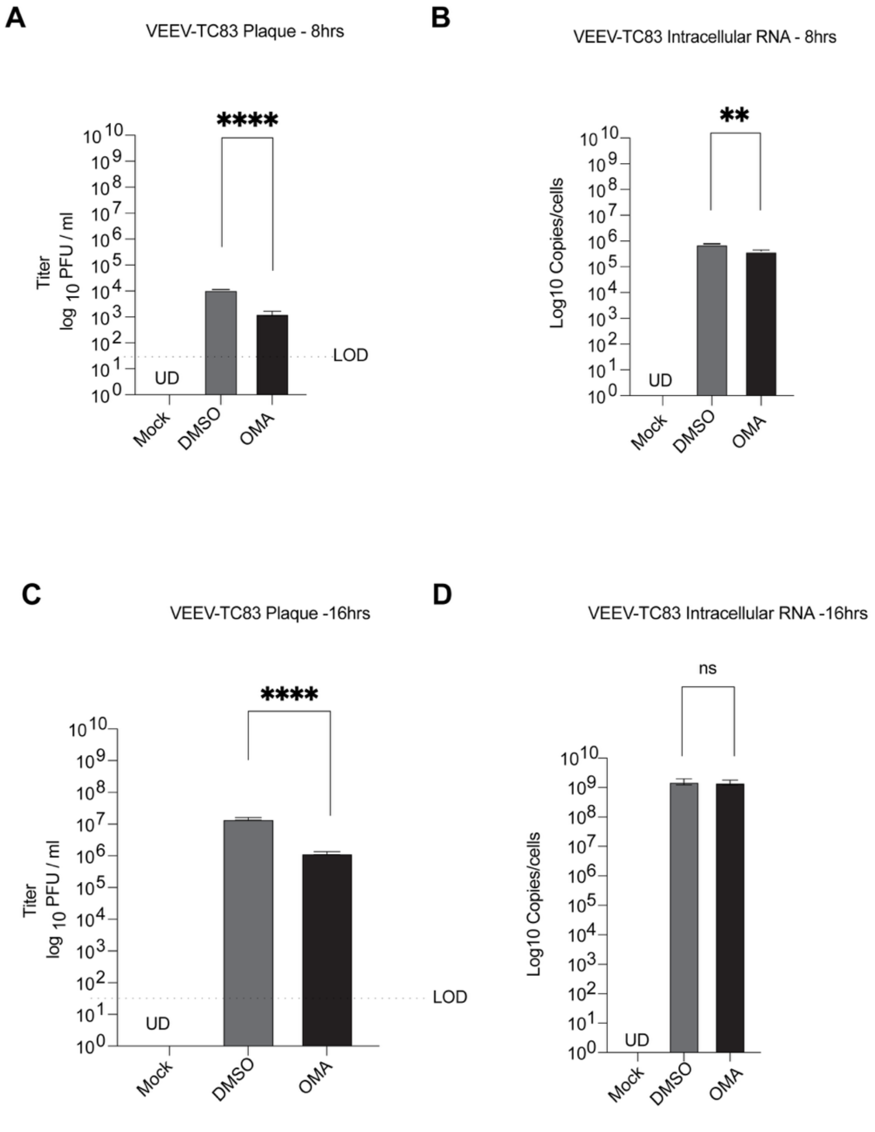

Supplemental Figure S5. Quantification of viral RNA via qRT-PCR of VEEV TC-83 infected LUHMES cells for gene expression analysis. Plaque viral titer quantification (**A**) and viral RNA quantification (**B**) assessing the efficacy of OMA on VEEV TC-83 in 6d diff. at 8hpi at an MOI of 0.1. Plaque viral titer quantification (**C**) and viral RNA quantification (**D**) assessing the efficacy of OMA on VEEV TC-83 in 6d diff. at 16hpi at an MOI of 0.1. Statistical analysis was done using an unpaired t-test: ** $p < 0.01$, ****$p < 0.0001$, and p = *ns* (not significant). Statistical analysis was based on the average of four biological replicates.

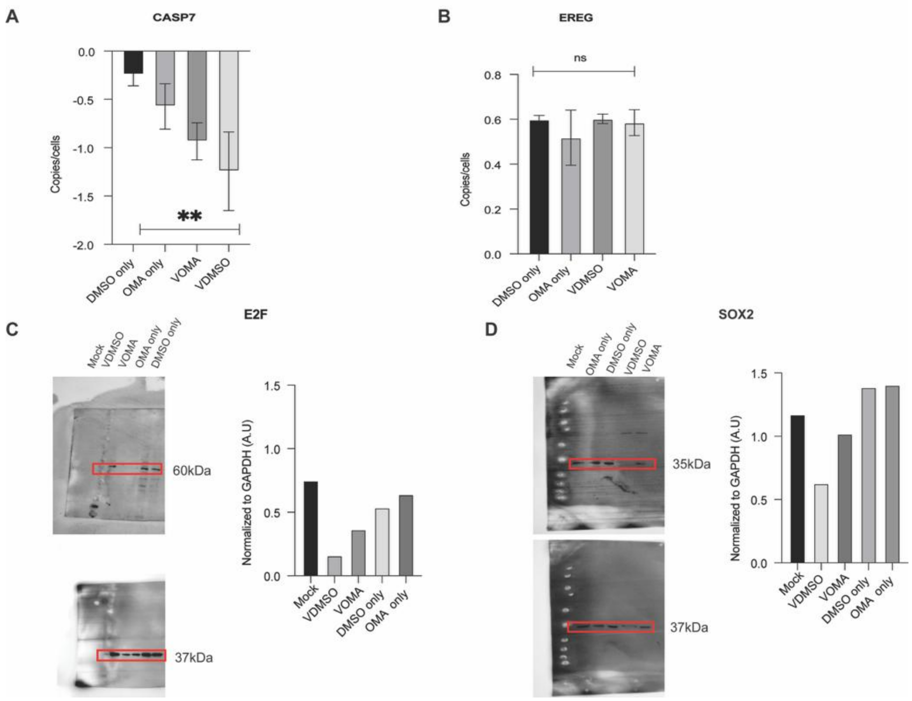

Supplementary Figure S6. Neurotoxicity and phosphosignaling validation of independent genes and protein expression. Quantitative RT-PCR analysis of **(A)** CASP7 (Caspase-7) and (**B**) EREG expression in cells treated with DMSO only, OMA only, VEEV-infected-OMA-treated (VOMA), or VEEV-infected-DMSO-treated (VDMSO). Gene expression was normalized to GAPDH and is presented as relative expression (copies/cells) compared with the mock control group. Western blots and corresponding quantification graphs of E2F protein (**C**) and SOX2 (**D**) expression. Whole-blot immunoblots are shown on the left, with protein band intensities quantified by normalization to GAPDH and expressed in arbitrary units (AU) for Mock, VDMSO, VOMA, DMSO only, and OMA only treatment groups. Statistical analysis was done using one-way ANOVA: ** *$p < 0.01$ and p = ns* (not significant). Analyses were based on three replicates for gene expression and one for protein expression.

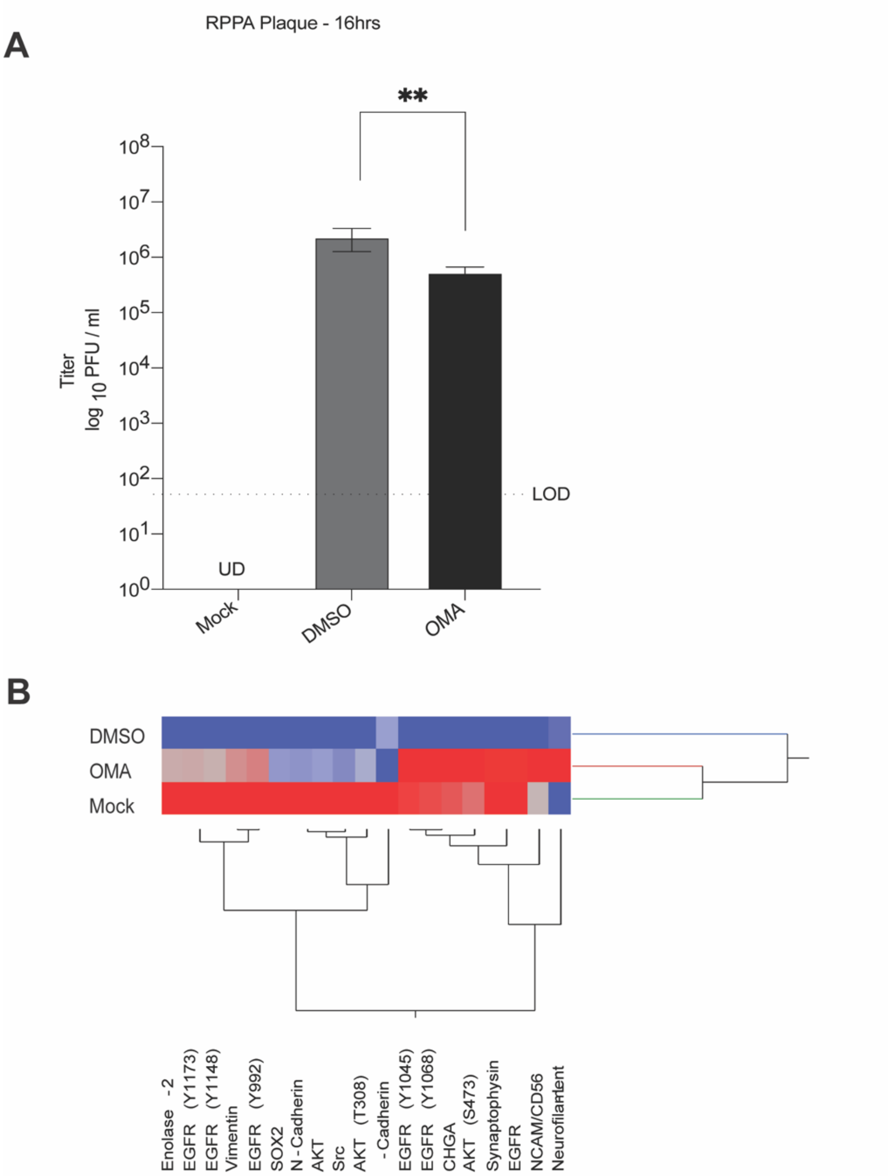

Supplemental Figure S7. Infectious virus titers and hierarchical clustering analysis from RPPA study. **(A)** Infectious virus titers in culture supernatants collected at 16 hours post-infection (hpi), quantified by plaque assay following infection at a multiplicity of infection (MOI) of 0.1. Statistical analysis using an unpaired t-test. ** $p < 0.01$ ($n = 3$). **(B)** Hierarchical clustering dendrogram illustrating the relationships between DMSO, OMA, and Mock samples. Statistical analysis was done using an unpaired t-test: ** *p < 0.01 using the average of 3 biological replicates.*

Supplementary Table S1. Antibodies for RPPA

Antibody	Catalog #	Vendor	RPPA Dilution	Host
Akt	9272	Cell Signaling	1:2000	R
AKT (S473) XP	4060	Cell Signaling	1:100	R
AKT (T308)	9275	Cell Signaling	1:100	R
CHGA	85798	Cell Signaling	1:100	R
E-Cadherin (24E10)	3195	Cell Signaling	1:400	RmAb
E2F	sc-251	Santa Cruz	1:500	R
EGFR	2232	Cell Signaling	1:100	R
EGFR (Y1045)	2237	Cell Signaling	1:50	R
EGFR (Y1068)	2234	Cell Signaling	1:50	R
EGFR (Y1148)	44-792G	Thermo Fisher	1:100	R
EGFR (Y1173)	44-794G	Thermo Fisher	1:100	R
EGFR (Y992)	2235	Cell Signaling	1:50	R
Enolase-2 (E2H9X) XP	24330	Cell Signaling	1:100	RmAb
FRS2-alpha (Y436)	3861	Cell Signaling	1:50	R
Gab1 (Y627)	3231	Cell Signaling	1:1000	R
GRB2	3972	Cell Signaling	1:1000	R
NCAM1 (CD56) (E7X9M)	99746	Cell Signaling	1:100	RmAb
6N-Cadherin	4061	Cell Signaling	1:200	R
Neurofilament-L (C28E10)	2837	Cell Signaling	1:100	RmAb
Rb (4H1)	9309	Cell Signaling	1:500	M
Rb (S780)	3590	Cell Signaling	1:2000	R
Rb (S807/811)	9308	Cell Signaling	1:200	R
Sox2	2748	Cell Signaling	1:100	R
Src (Y527)	2105	Cell Signaling	1:200	R
Synaptophysin (D8F6H)	36406	Cell Signaling	1:100	RmAb
Vimentin (D21H3)	5741	Cell Signaling	1:200	RmAb
Met (Y1234/1235)	3126	Cell Signaling	1:200	R
